# Revealing Evolutionarily Optimal Strategies in Self-Reproducing Systems via a New Computational Approach

**DOI:** 10.1007/s11538-019-00663-4

**Published:** 2019-11-18

**Authors:** Simran Kaur Sandhu, Andrew Morozov, Oleg Kuzenkov

**Affiliations:** 1 Department of Mathematics, University of Leicester, Leicester, UK; 2 Shirshov Institute of Oceanology, Moscow, Russia; 3 Lobachevsky State University of Nizhni Novgorod, Nizhni Novgorod, Russia

## Abstract

Modelling the evolution of complex life history traits and behavioural patterns observed in the natural world is a challenging task. Here, we develop a novel computational method to obtain evolutionarily optimal life history traits/behavioural patterns in population models with a strong inheritance. The new method is based on the reconstruction of evolutionary fitness using underlying equations for population dynamics and it can be applied to self-reproducing systems (including complicated age-structured models), where fitness does not depend on initial conditions, however, it can be extended to some frequency-dependent cases. The technique provides us with a tool to efficiently explore both scalar-valued and function-valued traits with any required accuracy. Moreover, the method can be implemented even in the case where we ignore the underlying model equations and only have population dynamics time series. As a meaningful ecological case study, we explore optimal strategies of diel vertical migration (DVM) of herbivorous zooplankton in the vertical water column which is a widespread phenomenon in both oceans and lakes, generally considered to be the largest synchronised movement of biomass on Earth. We reveal optimal trajectories of daily vertical motion of zooplankton grazers in the water column depending on the presence of food and predators. Unlike previous studies, we explore both scenarios of DVM with static and dynamic predators. We find that the optimal pattern of DVM drastically changes in the presence of dynamic predation. Namely, with an increase in the amount of food available for zooplankton grazers, the amplitude of DVM progressively increases, whereas for static predators DVM would abruptly cease.

**Electronic supplementary material:**

The online version of this article (10.1007/s11538-019-00663-4) contains supplementary material, which is available to authorized users.

## Introduction

Complex behavioural responses and sophisticated life history traits of individual organisms observed in the natural world should have a great influence on ecological processes (reproduction, competition, mortality, etc.), and we often need to incorporate them in our population and ecosystem models to be able to improve their forecasting power. Currently, there exist various modelling frameworks to reveal behaviours and life history traits emerged as a result of long-term evolution. For example, the conventional paradigmatic idea is that one needs to optimise a certain initially prescribed criterion such as the ratio between the growth and mortality rates of an organism (Gilliam and Fraser 1987; De Robertis 2002; Sainmont et al. 2015), the individual reproductive value (Mangel and Clark 1988; McNamara et al. 2001; Fiksen and Carlotti 1998), some generalised entropy function (Levich 2000) or some particular life history trait, for example mortality or the growth rate (Han and Straskraba 1998, 2001). Behavioural patterns can also be modelled via different game-theoretical approaches, where we assume that organisms maximise their gain described via a certain pay-off matrix which is related to some ecological rates (Hofbauer and Sigmund 1998; Broom and Rychtar 2013). The potential difficulty of the above approaches is that the choice of the criterion (or the corresponding pay-off matrix) which we need to optimise is often subjective and may entirely depend on the personal choice of the modeller (Morozov and Kuzenkov 2016).

The main alternative to the maximisation of a certain prescribed criterion is the use of underlying models of population dynamics, which is realised in several well-known approaches. For example, in the classical adaptive dynamics (based on the so-called canonical equation) the evolutionary outcome emerges as a result of a large number of consecutive small-sized and rare mutations, their further invasion and replacement of the resident population (Geritz et al. 1998; Kisdi and Geritz 2010; Kisdi and Priklopil 2011). Using adaptive dynamics, it was shown that under some rather strict assumptions about environmental feedback, the evolutionary outcome can be found by no more than maximisation of invasion fitness (Gyllenberg and Service 2011; Gyllenberg et al. 2011). Another class of generic modelling techniques known as genetic algorithms is designed to imitate long-time evolutionary dynamics including the processes of mutation, crossover, selection and replacement (Hamblin 2013; Sumida et al. 1990). Some advanced computational approaches include agent-based modelling (or individual-based modelling) by describing movement, competition and reproduction of each individual through space and time to be able to accurately predict evolution (Hellweger et al. 2016); however, due to high computational cost (even for modern computers) their practical application in large-scale ecosystem models is not clear (Sainmont et al. 2015; Hamblin 2013).

The choice of an appropriate modelling framework to reveal the evolutionarily optimal strategy would depend on the system complexity (e.g. particular species and the surrounding environment), time and space scales involved. On the other hand, we cannot often derive the optimal strategies analytically directly from model equations or theoretically prove convergence to optimal strategies in the course of evolution due to consecutive mutations (Parvinen et al. 2006): in this case, we should rely on numerical techniques. For example, we still need simple and reliable computational methods for obtaining optimal strategies in population models including several developmental stages and in the situation where the phenotype is determined by complex behaviour mathematically described by a set of functions (function-valued traits). Note that genetic algorithms of evolutionary modelling are generally inefficient to deal with function-valued traits since they simulate discrete-valued problems (Hamblin 2013). Finally, our methods should allow us to consider large (i.e. not only small) mutations in the genotype space.

Here, we suggest a new computational method of finding the evolutionarily optimal strategy/life history trait which is based on an approximation of evolutionary fitness derived from the underlying model equations of population dynamics. The method can be applied to population models for which a global fitness function exists (conditions of implementation of the method are formulated in Sect. 2). By considering long-term population dynamics of several competing subpopulations having different traits and ordering them according to their competitive ability, we can reconstruct the fitness function as a function of model parameters and eventually find the evolutionarily optimal behaviour as the maximal value of fitness subject to possible trade-offs. The method allows us to work both with scalar and function-valued traits. Interestingly, our approach can be applied in the case where we ignore the underlying model equations, i.e. by only using data on population dynamics.

As a meaningful biological illustration, we implement our computational method to find patterns of optimal diel vertical migration (DVM) of zooplankton in the ocean and lakes which is considered to be the largest synchronised movement of biomass on Earth (Hays 2003; Kaiser 2005). Using a stage-structured model with static and dynamic visual predators, we construct an optimal daily trajectory of zooplankton in the water column, which can be considered as a function-valued trait. We show that including a dynamic predator of zooplankton (e.g. the own predator biomass depends on the amount of zooplankton consumed) would produce different predictions of DVM as compared to the situation with static predators when the amount food (phytoplankton) available for zooplankton grazers in surface layers progressively increases. This confirms the importance of considering dynamical feedback from the environment in evolutionary modelling and highlights limitations of the conventional approach of modelling DVM of zooplankton via maximisation of the reproductive value (Fiksen and Carlotti 1998; Sainmont et al. 2015).

The paper is organised as follows. Section 2 introduces the general methodology of approximation of fitness function(al) for a population model with inheritance. We state the assumptions under which the fitness function can be constructed. In Sect. 3, we describe the model settings for the structured zooplankton population model with a dynamic predator, where ecological rates (reproduction, mortality, maturation) are functions of DVM. In Sect. 4, we give examples of optimal DVM trajectories constructed for the model introduced in Sect. 3 and explore their dependence on key model parameters. Finally, in Sect. 5, we discuss the general applicability of our method and consider its possible advantages.

## General Methodology

### Defining the Fitness Function

Consider a generic self-replicating system, where different species are described in terms of an inherited strategy (or a trait) *v* belonging to a certain space *V*. In particular, *v* can be a function-valued trait. We assume that *v* belongs to a compact domain in a metric space *V* equipped with a Borel measure $\mu ^*$. (The measure is required to rigorously quantify sets of hereditary strategies).

The presence of each strategy *v* in the system at time *t* is characterised by a non-negative quality $\rho (v,t)$ which is a continuous function of *t* and *v*. Thus, $\rho (v,t)$ is a function on the space *V* integrable with respect to measure $\mu ^*$. In the case where the strategy *v* is absent, we have $\rho (v,t) \equiv 0$ and if it is present in the system, we have $\rho (v,t) > 0$. Biologically, $\rho (v,t)$ can be the population size, biomass, population density or some functions of them, for instance, this can be a positive power of the population size. We postulate that the extinction of *v* will correspond to these characteristics approaching zero. We assume that the population is uniformly bounded, i.e. the integral of $\rho (v,t)$ over the space *V* is smaller than a certain positive constant.

For the sake of simplicity, we assume that our system has the property of strong (clonal) inheritance, i.e. the strategy *v* only produces offspring with the same strategy. Thus, the case $\rho (v,t_0) = 0$ would signify that $\rho (v,t) \equiv 0$ for all $t>t_0$. We will discuss the possibility of including mutations in our framework in Sect. 5.

Consider a typical situation where the dynamics of $\rho (v,t)$ depends on a finite set of parameters $\overrightarrow{M}(v)=(M_1(v),\ldots ,M_n(v))$, each of which is defined by the strategy *v* as a certain function (for a scalar life history trait) or a functional (for function-valued traits). The dynamics of $\rho (v,t)$ is given by

1 $$\begin{aligned} \frac{\partial \rho (v,t)}{\partial t}=F_{v}(\rho ,t,\overrightarrow{M}(v)), \end{aligned}$$

where $F_{v}(\rho ,t)$ is a certain function that will be specified by choosing a concrete population model (see Sect. 3 for a case study example).

We introduce the following definition of ranking ordering.

#### Definition 1

(*Ranking order*) We state $v\succ w$, that is, strategy *v* is more advantageous, or fitter, than strategy *w* if the ratio between the densities tends to zero uniformly in some vicinities *O*(*v*) and *O*(*w*) of *v* and *w*, i.e.

2 $$\begin{aligned} \lim _{t\rightarrow \infty }\frac{\rho (w',t)}{\rho (v',t)}=0, \forall v'\in O(v), w'\in O(w). \end{aligned}$$

For fixed initial conditions, Definition 1 establishes a partial ranking order in the space *V*; in other words, the introduced relation satisfies axioms of transitivity (from $v\succ w$ and $w\succ u$ it follows that $v\succ u$) and anti-reflexivity (an element cannot be better than itself). We should stress that the above-introduced order is a partial one, i.e. for some elements the limit in expression (2) can be a positive number; in this case, both strategies may coexist or may both go extinct. Note that here we focus on the scenario where the introduced ranking order does not depend on the initial condition. In this case, when randomly generating several strategies, the probability that some strategies show coexistence will be zero (for detail see next subsection). We also briefly consider a model study case where the ranking order can depend on the initial conditions (Sects. 2.3 and 4.2).

In the case of existence of uniform long-term average per capita rates $R_v$ and $R_w$ of strategies *u* and *v*, respectively, the condition $R_v>R_w$ guarantees $v\succ w$ (see Kuzenkov and Ryabova 2015a for details).

3 $$\begin{aligned} R_v= & {} \lim _{T\rightarrow \infty }\frac{1}{T}\int _{0}^{T}\frac{\rho _{t}(v,t)}{\rho (v,t)}\hbox {d}t =\Big \langle \frac{\rho _{t}(v,t)}{\rho (v,t)} \Big \rangle \nonumber \\> & {} \lim _{T\rightarrow \infty }\frac{1}{T}\int _{0}^{T}\frac{\rho _{t}(w,t)}{\rho (w,t)}\hbox {d}t= \Big \langle \frac{\rho _{t}(w,t)}{\rho (w,t)}\Big \rangle = R_w, \end{aligned}$$

where $\rho _{t}$ denotes differentiation with respect to *t* and convergence in the above limits is uniform in *V*; angle brackets denote time averaging. Note that $v\succ w$ does not necessarily imply $R_v>R_w$.

#### Assumption 1

We suppose that the introduced ranking order does not depend on initial conditions.

#### Remark

We should admit that Assumption 1 may be difficult to verify analytically for an arbitrary model. One can always apply extensive numerical simulations by exploring various combinations of initially present strategies and their initial densities; however, this can be computationally costly. Note that there are some classes of models where the existence of fitness independent on initial conditions can be justified (Kuzenkov and Morozov 2019). In particular, this includes single population models with stage/age structuring including both discrete and continuous approach (Kuzenkov and Morozov 2019) as well as the famous Lotka–Volterra model (Morozov and Kuzenkov 2016). Interestingly, for some models as the predator–prey model with a logistic prey growth (Sects. 2.3 and 4.2)—which have a frequency-dependent selection and Assumption 1 is not satisfied—we may still apply our computational method. This is explained in more detail in Sects. 2.3 and 4.2 as well as in the supplementary material.

From the above assumption, it is clear that the remaining strategy (or several strategies) will be the one which has the highest ranking with respect to the introduced order with nonzero initial densities.

The concept of maximisation of fitness which is fundamental for our method comes from the following assumption:

#### Assumption 2

We suppose that Assumption 1 holds. We also assume that the (minor) restrictions of the existence of a functional *Y*(*v*) reflecting the introduced ranking order hold (for detail on restrictions see Krantz et al. 1971), i.e. $Y(v)>Y(w)$ if $v\succ w$.

#### Remark

The function(al) *Y*(*v*) stated in Assumption 2 mathematically expresses population (evolutionary) fitness.

Temporal dynamics of $\rho (v,t)$ in equation(s) (1) are determined by parameters $\overrightarrow{M}=(M_1(v),\ldots ,M_n(v))$, which are function(al)s of strategy *v*, thus *Y* will be a multi-variable function of $\overrightarrow{M}$, i.e. $Y(v) \equiv J(\overrightarrow{M(v)})$. In the case where we have an analytical expression for *Y* as a function of $\overrightarrow{M}$ and we also know mathematical formulations of $\overrightarrow{M}$ as function(al)s of *v*, we can fully determine the fitness *Y* as a function(al) on space *V* and finally determine the evolutionarily optimal strategy which maximises this functional.

**Fig. 1:**
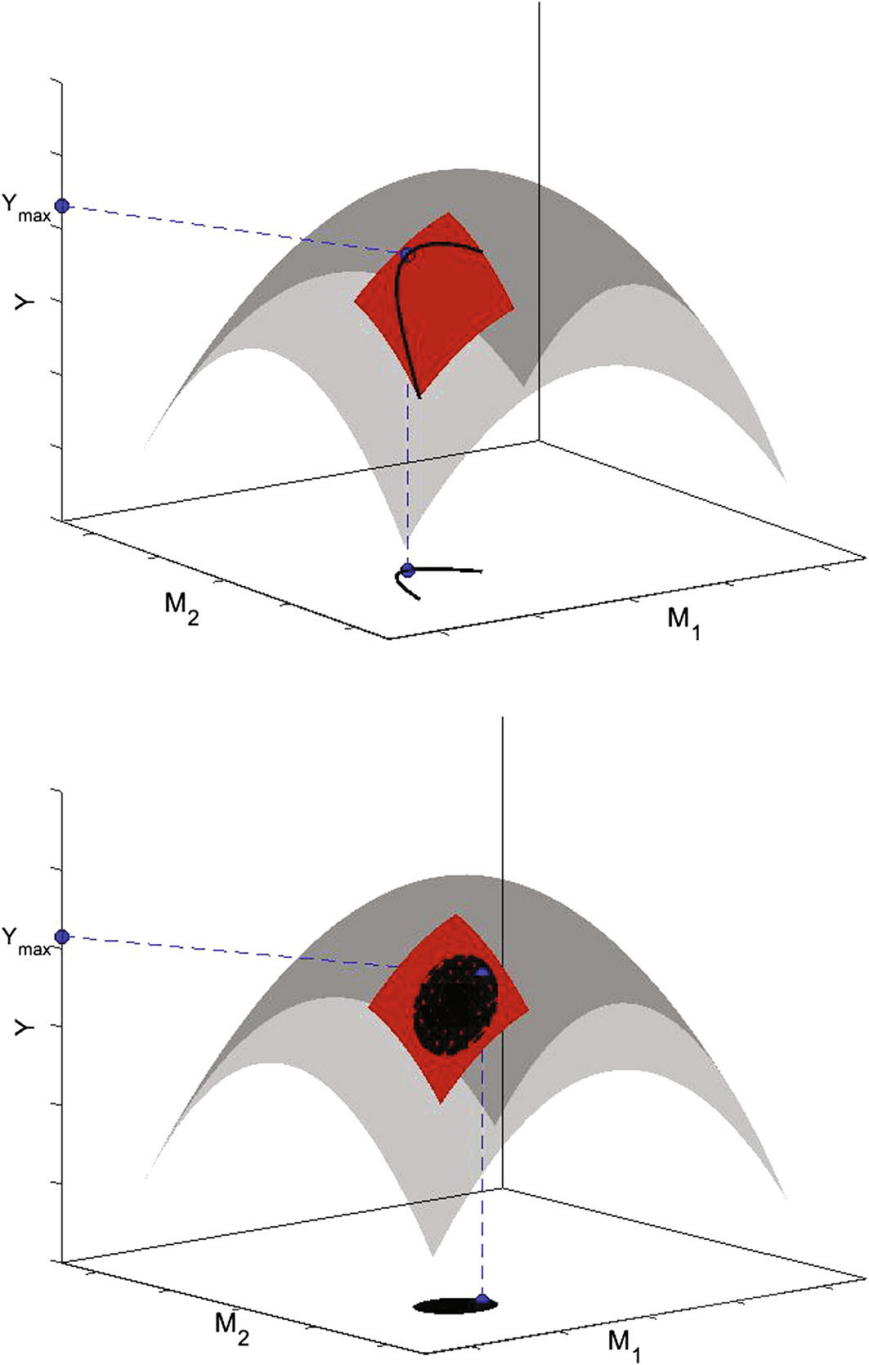
Fitness function *Y* plotted as a multi-variable function of model parameters *M*: a higher value of *Y* signifies a higher competitive order. The maximal value of fitness (under constraints on the domain) is denoted by $Y_\mathrm{max}$. Upper panel: values of parameters are related via a trade-off mechanism. Lower panel: possible values of *M* are continuously distributed within a certain compact domain

The concept of evolutionary fitness is explained in Fig. 1, where *Y* is assumed to depend on 2 parameters *M*, for simplicity. Increasing the value of *Y* would correspond to transition to better strategies. On the other hand, the global maximum of $Y(\overrightarrow{M})$ would not necessarily correspond to the best possible strategy in the system. This is the case where parameters are related by some trade-off dependence and/or are bounded (see Fig. 1a). In the case, where *v* is a function (or components $\overrightarrow{M}$ are not related by trade-offs), the overall span of strategies *V* can generate a bounded domain in space $\overrightarrow{M}$ which might not contain the global maximum of $Y(\overrightarrow{M})$, thus the optimal strategy is achieved at the boundary (Fig. 1b).

In practice, finding the fitness function (provided such a function exists for the given model) consists of reconstructing or approximating the shape of *Y* as a function of model parameters $\overrightarrow{M}$ by considering a limited number of competing strategies in a way that the approximation of *Y* would preserve the introduced ranking order of strategies. To be able to do that efficiently we make the final assumption.

#### Assumption 3

The function $Y(\overrightarrow{M})$ is a sufficiently smooth function (*k* times differentiable) of model parameters $\overrightarrow{M}$.

Under the above assumption, we can approximate *Y* via the following Taylor expansion around a certain point $M_0$ which is assumed to be close to the optimal strategy $M(v^{*})$ but does not necessarily coincide with it.

4 $$\begin{aligned} Y(\overrightarrow{M})&=Y(\overrightarrow{M_0})+DY(\overrightarrow{M_0})\overrightarrow{\delta M} +\frac{1}{2}\overrightarrow{\delta M}D^2J(\overrightarrow{M_0}) (\overrightarrow{\delta M})^T+\cdots + o(\Vert \overrightarrow{\delta M}\Vert ^k)\nonumber \\&=\hbox {const} + \sum _{i=1}^{n}B_{i}M_{i}+\sum _{i=1}^{n}\sum _{i\le j}^{n}C_{ij}M_{i}M_{j}+\cdots + o(\Vert \overrightarrow{\delta M}\Vert ^k). \end{aligned}$$

Thus, to find evolutionary fitness we need to estimate the coefficients $B_i$, $C_{ij}$, etc., in the above Taylor expansion. We can also remove the constant since we are interested not in finding the absolute value of the maximum of *Y* but in obtaining the strategy $v^*$ which realises this maximum.

The generic algorithm of approximating fitness function *Y* and finding the evolutionarily optimal strategy is provided in the next section.

### The Algorithm of Computing the Evolutionarily Optimal Strategy

Our algorithm consists of the four steps discussed in detail below.

#### Step 1: Approaching the Vicinity of the Maximal Fitness 

Although Assumption 3 allows us to consider as many terms in the Taylor series (4) as possible, for practical reasons it is convenient to stick to the quadratic or even to the linear part. In this case, it is important to stay in the vicinity of the optimal strategy. The main goal of Step 1 is to approach/localise the vicinity of the maximum point of *Y*, and there exist different ways to achieve this goal.

In this paper, we use Monte-Carlo simulations (Zhigljavsky and Zilinskas 2008). We generate random parameters $\overrightarrow{M}$ (by generating an everywhere dense sequence of points) taking into account possible constraints on parameters and trade-offs. Among them are obvious requirements of positivity for the growth rates, mortality terms, etc. However, a more complicated situation arises in the case where *v* is a function and its variation would result in a restriction in terms of $\overrightarrow{M(v)}$. To overcome this difficulty, one can first estimate the boundary of the domain of feasible parameters $\overrightarrow{M}$ by varying functions *v*, for example by considering different combinations in few first coefficients of the Fourier (or Taylor) series (see an illustrative example in SM4).

For the randomly generated points $\overrightarrow{M}$, we simulate their joint long-term dynamics using (1). Then, we rank competing strategies based on their per capita growth rates, and then, using this ranking we approximate the location of the best point with the maximal fitness. Here, we assume that we do not have the situation where strategies coexist, i.e. to have strategies with equal fitness. This signifies that we do not have neutral evolution, where a set of points of equal fitness in the considered *n*-dimensional parametric space may have a nonzero measure. For example, for $n=2$ the curves of equal fitness should be smooth.

We only need to find the vicinity where our approximation of fitness in the next steps becomes satisfactory (this can be checked by the implementation of the procedures from the next steps). To avoid potentially missing the global maximum and sticking to a local one, we need to take a sufficient number of points for Monte-Carlo simulations, which can be estimated based on the size of the domain and the required accuracy (e.g. SM 2). We should stress, however, that there exists no general recommendation about choosing the number of points since this would strongly depend on a particular fitness function. (Obviously, for a function with a sharp maximum, one will need more points.)

We should stress that choosing a sufficiency large number of points in Step 1 would potentially allow us to find the optimal parameters without proceeding to further steps. However, in the case of a large number of parameters and in the situation where the strategy *v* is a function (or a vector of functions) with a complicated shape probably including discontinuity, the required number of random points to accurately approximate such a strategy will be extremely large (e.g. exceeding computer memory). In this case, we will need to proceed to a semi-analytical approach given by steps 2 and 3.

#### Step 2: Approximating the Fitness Function

Once we have a set of strategies that are close to the optimal one, we can approximate fitness *Y* using (4). We consider those *N* strategies which are most closely located to the optimal strategy (using the results of simulations obtained in step 1). We rank the strategies based on their long-term average per capita growth rates. Let us denote already ordered strategies by $j=1,2,\ldots , N$ and their respective per capita growth rates by $R_j$. For the growth rates, we have the following inequalities $R_1> R_2> R_3>\cdots > R_N$ which is equivalent to the system of $N-1$ inequalities for the fitness function *Y*

5 $$\begin{aligned} \begin{array}{c} Y(\overrightarrow{M_N})-Y(\overrightarrow{M}_{N-1})<0,\\ \vdots \\ Y(\overrightarrow{M_3})-Y(\overrightarrow{M_2})<0,\\ Y(\overrightarrow{M_2})-Y(\overrightarrow{M_1})<0. \end{array} \end{aligned}$$

We can use our approximation of fitness to obtain the inequality conditions for the coefficients $B_i$, $C_{ij}$ in Eq. (4). We reduce the number of unknown coefficients by setting one of the coefficients to be 1; for example, we can take $B_{1}=1$ or, alternatively, we can require that the sum of all coefficients be 1. Therefore, system (5) can be reduced to

6 $$\begin{aligned} \begin{array}{c} \overrightarrow{B} ( \overrightarrow{M}_{N}-\overrightarrow{M}_{N-1})+ \overrightarrow{M}_{N} C ( \overrightarrow{M}_{N})^T-\overrightarrow{M}_{N-1} C ( \overrightarrow{M}_{N-1})^T<0,\\ \vdots \\ \overrightarrow{B} ( \overrightarrow{M}_{3}-\overrightarrow{M}_{2})+ \overrightarrow{M}_{3} C ( \overrightarrow{M}_{3})^T-\overrightarrow{M}_{2} C ( \overrightarrow{M}_{2})^T<0, \\ \overrightarrow{B} ( \overrightarrow{M}_{2}-\overrightarrow{M}_{1})+ \overrightarrow{M}_{2} C ( \overrightarrow{M}_{2})^T-\overrightarrow{M}_{1} C ( \overrightarrow{M}_{1})^T <0. \end{array} \end{aligned}$$

The above inequalities provide estimates for $B_i$ and $C_{ij}$. In fact, any set of coefficients satisfying the system of equations (6) can be chosen as its solution. This uncertainty can be reduced by taking the number *N* of generated strategies to be high; in this case, the size of the domain restricted by the system of equations (6) would shrink, thus choosing any coefficients satisfying the system would provide similar results for the approximation of *Y*. On the other hand, the use of a large number of strategies *N* may cause the system to have no solution, i.e. to be inconsistent. This can occur since the quadratic approximation does not coincide with the true fitness function which has infinitely many Taylor terms. To obtain $B_i$, $C_{ij}$, in this case, one can consider higher order terms in the approximation of *Y* or reduce the size of the domain. Technically, system (6) is solved via the Simplex method by using standard software (e.g. MATLAB), where the objective functional can be taken as a certain arbitrary linear function of coefficients; for example, this can be the sum of the $B_i$s. However, one can also use some more advanced method, for example, the Nelder–Mead simplex algorithm.

#### Step 3: Finding the Optimal Fitness

After completion of the previous steps, we arrive at the following approximation of *Y*

7 $$\begin{aligned} Y(v) \approx \sum _{i=1}^{n}B_{i}M_{i}(v)+\sum _{i=1}^{n}\sum _{i\le j}^{n}C_{ij}M_{i}(v)M_{j}(v), \end{aligned}$$

where fitness is now considered as a function(al) depending on strategy *v*. We can now search for the best strategy $v^*$ which maximises expression (7) and belongs to space *V*. The advantage of using the above quadratic (or sometimes linear) form is that we can solve the optimisation problem semi-analytically even if *v* is a function (which was not possible using the approximation $\overrightarrow{G}$ in step 1). The technique of solving the optimisation problem semi-analytically (related to the model in Sect. 3) is briefly described in Supplementary material SM3.

#### Step 4: Increasing the Accuracy of our Solution (Optional)

After finding the approximation of the best strategy $v^*$ in step 3, we may check (if needed) the accuracy by generating some additional strategies in the vicinity of this point and repeating steps 1–3. We expect the convergence of the method for a sufficiently smooth fitness function since the overall ideology of the method is similar to that of gradient methods (with a vast literature existing on the topic).

### Constructing evolutionary fitness depending on initial conditions (an insightful example)

The above numerical algorithm suggests the independence of fitness from initial conditions (as stated in Assumptions 1 and 2) and this restricts a straightforward implementation of the method. We should say, however, that in some cases the algorithm can be implemented even if fitness and the ranking order depend on initial conditions. Below, we provide an insightful example where fitness can be numerically constructed for a model where Assumptions 1 and 2 do not hold. (Similar principles are used to deal with the more complicated predator–prey model in Sect. 4.2.) However, we must stress that this should be only understood as a starting point of research (rather than an exhaustive study) which demonstrates that the method allows extensions beyond Assumptions 1 and 2.

Consider the following classical predator–prey model with multiple prey species and a single predator. Here, *V* is the space of possible strategies *v* of prey, *z*(*v*, *t*) is the prey density corresponding to the strategy *v*, and *F*(*t*) is the population size of the predator. The model equations for *z* and *F* are given by

8 $$\begin{aligned} z_{t}(v,t)= & {} r(v) z(v,t) - c(v) z(v,t) F(t)- z(v,t)\int _{V}z(v,t) \mu ^*(\hbox {d}v) , \end{aligned}$$

9 $$\begin{aligned} F_{t}(t)= & {} F(t)\int _{V}c(v) z(v,t) \mu ^*(\hbox {d}v) -F(t), \end{aligned}$$

where *r* and *c* are, respectively, the reproduction coefficient and the attack rate of the predator. For simplicity, the mortality rate of predator and the food conversion coefficient are set to be one. We consider that the strategy *v* determines the values of *r* and *c*; we can consider the space *Q* consisting of pairs (*r*, *c*). In the above equations, the measure $\mu ^*$ can be understood as the classical Lebesgue measure meaning the area in *Q*. However, we can also consider $\mu ^*$ to be concentrated in a finite set of points (atomic measures): in this case, the above integrals will transform into sums over a finite number of genotypes.

For the above model, the ranking order given by Definition 1 depends on initial conditions; in particular, it will be determined by the set $Q_0$, where $z(c,r,0)>0$. Indeed, the average per capita rate for *z*(*c*, *r*, *t*) is defined as

$$\begin{aligned} \Big \langle \frac{z_{t}(c,r,t)}{z(c,r,t)} \Big \rangle =r - c \big \langle F(t) \big \rangle - \Big \langle \int _{Q}z(c,r,t) \mu ^*(\hbox {d}v) \Big \rangle , \end{aligned}$$

which is equivalent to the following expression

$$\begin{aligned} \Big \langle \frac{z_{t}(c,r,t)}{z(c,r,t)} \Big \rangle =r - c \big \langle F(t) \big \rangle . \end{aligned}$$

Here, the long-term average value of *F*(*t*) (shown in angle brackets) is determined based on the survived prey. (Technically, it can be found by calculating the equilibrium values.) Let us consider the situation where only one strategy survives (denoted by $(c_0,r_0)$). We have

$$\begin{aligned} \Big \langle F(t) \Big \rangle =\frac{r_0}{c_0}- \frac{1}{c_0^2}. \end{aligned}$$

We now introduce the following function of four arguments

10 $$\begin{aligned} Y_1(c,r,c_0,r_0)= r-c\Big ( \frac{r_0}{c_0}-\frac{1}{c_0^2} \Big ), \end{aligned}$$

which we will call the fitness associated with the strategy $(c_0,r_0)$. Note that this function is continuous in the compact set $Q\times Q=Q^2$. The strategy $(c_0,r_0)$ will be the best in $Q_0$ with respect to any other strategy if for any (*c*, *r*) from $Q_0$ we have

11 $$\begin{aligned} Y_1(c,r,c_0,r_0)= r-c\Big ( \frac{r_0}{c_0}-\frac{1}{c_0^2} \Big ) < r_0-c_0\Big ( \frac{r_0}{c_0}-\frac{1}{c_0^2} \Big )=\frac{1}{c_0} =Y_1(c_0,r_0,c_0,r_0) . \end{aligned}$$

For a different $Q_0$, the best strategy will be in general different and this will change both the fitness function and the associated ranking order.

Another scenario assumes the survival (with further mutual coexistence) of more than one strategy. It can be easily seen that for survival of two strategies $(c_1,r_1)$ and $(c_2,r_2)$, it is required that

$$\begin{aligned} r_1-c_1\Big ( \frac{r_2}{c_2}-\frac{1}{c_2^2} \Big )> \frac{1}{c_2}, r_2-c_2\Big ( \frac{r_1}{c_1}-\frac{1}{c_2^2} \Big ) > \frac{1}{c_1} , \end{aligned}$$

In other words, it is required that $Y_1(c,r,c_2,r_2)>Y_1(c_2,r_2,c_2,r_2)$ and $Y_1(c_2,r_2,c_1,r_1)>Y_1(c_1,r_1,c_1,r_1)$. Simple computation gives $\langle F(t)\rangle =(r_2-r_1)/(c_2-c_1)$. We can now use the following function associated with the strategies $(c_1,r_1)$ and $(c_2,r_2)$

$$\begin{aligned} Y_2(r,c,r_1,c_1,r_2,c_2)=r-c\frac{r_2-r_1}{c_2-c_1}, \end{aligned}$$

In the case of survival of two different strategies, for all other strategies in $Q_0$ we should have $Y_2(r,c,r_1,c_1,r_2,c_2)<Y_2(r_1,c_1,r_1,c_1,r_2,c_2)=Y_2(r_2,c_2,r_1,c_1,r_2,c_2)$.

Note that survival of two distinct strategies becomes impossible if the set *Q* is strictly convex. Indeed, in a strictly convex set there will always be some points located on both sides of the line connecting the points $(c_1,r_1)$ and $(c_2,r_2)$ (which we assume to be the best) and there will be always a point (*c*, *r*) such that $Y_2(r,c,r_1,c_1,r_2,c_2)>Y_2(r_1,c_1,r_1,c_1,r_2,c_2)=Y_2(r_2,c_2,r_1,c_1,r_2,c_2)$ which contradicts our assumption that $(c_1,r_1)$ and $(c_2,r_2)$ are the best (and the only surviving) strategies. Note that survival of three (or more) strategies in this model can only occur if $Y_2(r,c,r_1,c_1,r_2,c_2)=Y_2(r_1,c_1,r_1,c_1,r_2,c_2)=Y_2(r_2,c_2,r_1,c_1,r_2,c_2)$ which is structurally unstable: any small perturbation of $Q_0$ results in extinction of a strategy.

We will further assume that the set *Q* is strictly convex: in this case, evolutionary fitness will be given by the function $Y_1(c,r, c^*,r^*)$ defined by (10) where $(c^*,r^*)$ is the best (and unique) point of *Q*. For we have $Y_1(c^*,r^*, c^*,r^*)= \max _{Q}Y_1(c,r, c^*,r^*)$. Note that the fitness defined by $Y_1(c,r, c^*,r^*)$ generally depends on initial condition. Our main goal is to estimate $(c^*,r^*)$. Below, we show that the numerical algorithm suggested in Sect. 2.2 remains valid for the given model (as well as for the extension of this model considered in Sect. 4.2).

Indeed, Step 1 of the method consists in covering of the set *Q* by a set of $Q_N$ of *N* randomly chosen points with a further simulation of joint long-term dynamics using (8)–(9). As a result, one can determine the best point $(c_0,r_0)$ in $Q_N$ which will correspond to the maximum of $Y_1(c,r,c_0,r_0)$ across the given set of finite points. The accuracy can be improved by adding a larger number of points as in the standard Monte-Carlo method. The proposition below provides the theoretical basis for the use of Monte-Carlo method for reaching the vicinity of the best point $(c^*,r^*)$ for a sufficiently large *N*.

#### Proposition 1

We assume that in model (8)–(9) the feasible (allowed) domain *Q* of parameters (*c*, *r*) is strictly convex. Generating a sequence of points which is everywhere dense will eventually (with a probability of one) result in the landing of at least one point into any $\epsilon$ vicinity of the best point $(c^*,r^*)$ of the maximal evolutionary fitness.

The proof of the above proposition is given in the supplementary material (SM1). The proposition states that by generating a sufficiently large number of points *N*, we will approach the best point $(c^*,r^*)$ with any required accuracy $\epsilon >0$. The average critical number of points $N_{cr}$ can be easily estimated (see SM2 for detail). Note that estimate for the $N_{cr}$ provided in SM2 has a straightforward extension to be implemented to the more complicated model in Sect. 4.2 with stage structuring in the prey population.

Proposition 1 solves the problem of localisation of the best point in the model (Step 1) in the space of model parameters. Further steps of the computation approach (similar to those in Sect. 2.2) will be needed to find a more accurate approximation of $(c^*,r^*)$ and eventually the best element $v^*$ in the underlying space of strategies *V*. Note that in the given model, the fitness function will be linear (in terms of *c* and *r*) and we can efficiently use optimisation techniques involving various gradient methods which results in a fast convergence. (The convergence of gradient methods is largely discussed in the literature.)

## Modelling Diel Vertical Migration of Zooplankton

Here, we apply the above method to a particular ecological case study, which is the regular daily vertical migration of zooplankton.

### Diel Vertical Migration (DVM) of Zooplankton

Regular diel vertical migration (DVM) of zooplankton is often considered to be the largest synchronised movement of biomass on our planet (Hays 2003; Kaiser 2005). It was reported that DVM greatly affects the biogeochemical cycles in the ocean, as they impact the vertical transport of microparticles and some dissolved gases (Bianchi 2007) serving as an efficient carbon pump (Hansen and Visser 2016). Typically, the pattern of DVM consists of organisms ascending to surface waters rich in phytoplankton for feeding at night, then descending to deeper depths and remaining there during the day (Ohman 1991; Hays 2003). It is currently believed that zooplankton performs DVM to avoid visual predation (by planktivorous fish) by spending daylight hours in the deeper and darker areas and migrating up at night when visual predators cannot see them (Ohman 1991; Lampert 1989; Fortier et al. 2001; Pearre 2003). However, along with the predator avoidance hypothesis, some other reasons for DVM have been cited, such as saving energy in deep waters due to the lower temperatures or avoiding solar radiation (Mangel and Clark 1988; Pearre 2003). Despite the phenomenon of DVM being extensively studied, we still have a poor understanding of the key factors that DVM depends on (Pearre 2003; Ringelberg 2003).

The existing models of DVM assume that the optimal behaviour would signify maximisation of a certain function which is postulated a priori in the literature. For example, this can be the expected individual reproductive value (Mangel and Clark 1988; Fiksen and Carlotti 1998; Sainmont et al. 2015), the ratio of energy gain/mortality known as Gilliam’s rule (De Robertis 2002; Sainmont et al. 2015; Hansen and Visser 2016) or the so-called ‘venturous revenue’ (Liu et al. 2003, 2006), which is the product of the food intake and the survival probability. In some models of DVM, it is postulated that the organisms should instantaneously minimise their predation pressure (Han and Straskraba 1998, 2001). Other modelling approaches use game theory stating that organisms should maximise their gain described by a certain pay-off matrix; however, the choice of such matrices can vary from model to model (Gabriel and Thomas 1988; Iwasa 1982). The major shortcoming of the above approaches is fixing a priori a specific rule/function which we further need to maximise, which seems to ignore the dynamic feedback between the environment and the zooplankton population dynamics. Here, we implement our new computational method to find optimal trajectories of DVM from the underlying equations for population dynamics.

### Population Dynamics Equations

We explore patterns of DVM in zooplankton using a generic predator–prey model with structured prey: in our model, the prey is zooplankton and the predator is planktivorous fish. For simplicity, we only include two stages of zooplankton which we refer to as adults and juveniles and use the same model structure as in Kuzenkov and Kuzenkova (2012), Morozov and Kuzenkov (2016). The model can be easily extended by including more developmental stages of herbivorous zooplankton. The model equations read

12 $$\begin{aligned} A_{t}(v,t)= & {} p(v)J(v,t)-s(v)A(v,t)-\phi _{1}A(v,t)I(t)-f_{A}(v)A(v,t)F(v,t), \end{aligned}$$

13 $$\begin{aligned} J_{t}(v,t)= & {} b_{0}(v)A(v,t)-p(v)J(v,t)-q(v)J(v,t)\nonumber \\&-\phi _{2}J(v,t)I(t)-f_{J}(v)J(v,t)F(v,t), \end{aligned}$$

14 $$\begin{aligned} F_{t}(v,t)= & {} -m_{F}F(v,t)+eF(v,t)\int _{v}(f_{A}(v)A(v,t) +f_{J}(v)J(v,t))\mu ^*(\hbox {d}v),\nonumber \\ \end{aligned}$$

where *A*(*v*, *t*) and *J*(*v*, *t*) are the densities of adults and juveniles, respectively, in the subpopulation using strategy *v* of DVM and *F*(*t*) describes the population density of the predator. In our previous terminology (see Sect. 2), the set of parameters $\overrightarrow{M}$ which depend on DVM strategies *v* is given by ($p,s,q,b_0$); the other parameters are considered to be strategy-independent.

The integral term $I(t)=\int _{v}(\theta J(v,t)+\kappa A(v,t))\mu ^*(\hbox {d}v)$ represents the intraspecific competition of subpopulations across possible strategies and that of adults and juveniles, where integration is done across all possible strategies; $\phi _{1}$ and $\phi _{2}$ are introduced to describe the different levels of competition for adults and juveniles. The weights $\theta$ and $\kappa$ describe the relative contributions of juveniles and adults in competition.

The reproduction by adults is described by the term $b_{0}(v)A(v,t)$ in Eq. (12), with $b_{0}$ being the reproduction coefficient. The term *p*(*v*)*J*(*v*, *t*) in Eqs. (12) and (13) gives the transition rate from juveniles into adults. The terms *s*(*v*) and *q*(*v*) are the mortality rates of adults and juveniles due to natural reasons and consumption by other visual predators which are not included in *F*. We assume that the density of these predators is constant, and we call them ‘static’ predators in contrast to the dynamic predator *F*.

The predation is modelled via the linear (i.e. Holling type I) functional response $f_{J}(v)J(v,t)F(v,t)$ and $f_{A}(v)A(v,t)F(v,t)$, where the coefficients $f_{J}$ and $f_{A}$ model the corresponding predator attack rates. However, one can extend the same system by considering more complicated functional responses with saturation. We consider the generic scenario where the predator can consume both adult and juvenile zooplankton grazers. $m_{F}$ is the rate of mortality of the predator, and *e* is the food conversion coefficient.

### Specification of Model Coefficients

In our model, strategy *v* describes a periodic change of depth by adults and juveniles $v=(x_A(\tau ), x_J(\tau ))$ that the zooplankton takes every 24 h due to DVM, where $\tau =0$ corresponds to 6am, $\tau =0.5$ corresponds to 6pm, and $\tau =1$ corresponds to 6am the following day. The depth of migration is scaled following (Morozov and Kuzenkov 2016), where an average depth of 0 corresponds to the middle of the euphotic zone, i.e. a positive depth represents shallower food-rich waters, whereas a negative depth represents deeper darker waters.

In parameterising the model, we follow (Morozov and Kuzenkov 2016). The reproduction is given by

$$\begin{aligned} b_{0}=\epsilon _{A}\int _{0}^{1}(\alpha _{A}(\tanh (\sigma x_{A})+1)-\beta _{A}(x_{A}^{'})^{2}-\delta _{A}\cosh (\xi x_{A}))\hbox {d}\tau , \end{aligned}$$

where $\epsilon _{A}$ describes the conversion rate of food gain into the newly produced juveniles, $\beta _{A}$ is the metabolic cost of migration (we suggest energy losses are due to resistance forces, Fiksen and Giske 1995), $\alpha _{A}$ is proportional to the amount of food available for adults, and $\sigma$ characterises the steepness of the vertical gradient in the distribution of food. The function $\tanh (\sigma x)+1$ mimics a typical vertical distribution of phytoplankton: it is highest in surface waters and becomes very low in deep waters. The last term describes losses in fecundity in very deep waters (due to low temperatures or oxygen depletion) as well as when approaching surface waters; $\xi$ characterises the width of the habitat; $\delta _{A}$ gives the strength of the fecundity loss.

The mortality terms *s* and *q* are parameterised as follows:

$$\begin{aligned} s= & {} \int _{0}^{1}\gamma _{A}(\tanh (\sigma x_{A})+1)(\sin (2\pi \tau )+1)\hbox {d}\tau ,\\ q= & {} \int _{0}^{1}\gamma _{J}(\tanh (\sigma x_{J})+1)(\sin (2\pi \tau )+1)\hbox {d}\tau . \end{aligned}$$

Here, we follow the assumption from Morozov and Kuzenkov (2016) that the vertical distribution of the ‘static’ predators of zooplankton follows the same distribution as that of phytoplankton; however, this assumption is not essential. We consider that the mortality caused by visual predators follows the variation of light intensity throughout the day. In the above expressions, $\gamma _{A}$ and $\gamma _{J}$ are products of the density of static predators and the attack rates.

The parameter *p* with the meaning of the inverse time of transition from juveniles to adults is described by a Monod-like equation

$$\begin{aligned} p=\frac{\eta L}{L+k} \end{aligned}$$

with *L* being the energy gain per day by the juveniles given by

$$\begin{aligned} L=\int _{0}^{1}(\alpha _{J}(\tanh (\sigma x_{J})+1)-\beta _{J}(x_{J}^{'})^{2}-\delta _{J}\cosh (\xi x_{J}))\hbox {d}\tau , \end{aligned}$$

where $\beta _{J}$ has the same meaning as $\beta _{A}$ in the above expression for $b_0$, $\delta _{J}$ describes losses of energy in very deep and surface waters, $\eta$ is the highest transition rate, and *k* is the half saturation constant.

The dependence of attack rates by the dynamic predator on time of day is given by

$$\begin{aligned} f_{A}= & {} \int _{0}^{1}\gamma _{F_{A}}(\tanh (\sigma x_{A})+1)(\sin (2\pi \tau )+1)\hbox {d}\tau ,\\ f_{J}= & {} \int _{0}^{1}\gamma _{F_{J}}(\tanh (\sigma x_{J})+1)(\sin (2\pi \tau )+1)\hbox {d}\tau , \end{aligned}$$

where $\gamma _{F_{A}}$ and $\gamma _{F_{J}}$ are the predation attack rates for adults and juveniles, respectively.

We used the same range of model parameters $\alpha , \beta , \gamma , \delta$ as in Morozov and Kuzenkov (2016) to be able to compare the previous results with those obtained via the new computational method. We approximate the trajectories in $(x_A(\tau ), x_J(\tau ))$ using the two first terms of Fourier series. Our simulation shows that for the considered values of parameters (see the next section) adding extra terms in Fourier series results in only a small improvement of about 2%.

## Revealing Optimal Trajectories of Zooplankton DVM

We will separately consider two scenarios: (i) static predation on the zooplankton (the level of predation does not depend on the population densities of the zooplankton) and (ii) dynamic predators which density depends by the amount of zooplankton consumed. Note that in case (i) Assumptions 1 and 2 are satisfied, whereas in case (ii) is not generally true.

### DVM Under the Static Predator Scenario

We set $F \equiv 0$ and include static predators in coefficients *s*(*v*) and *q*(*v*). Ecologically, this describes situations where the predator (planktivorous fish) density is mostly controlled by higher predator levels (e.g. piscivorous fish), or static predators can be generalists, where migrating zooplankton species constitute only a small part of their diet. Note that for $F \equiv 0$ Assumptions 1 and 2 are satisfied and we can implement the computational method.

We start with a comparison of our approximation of fitness using the new computational method and a simple analytical expression for fitness derived in Morozov and Kuzenkov (2016), for a particular case where $\phi _{1}=\phi _{2}$ (Morozov and Kuzenkov 2016)

$$\begin{aligned} Y(v)=-s(v)-p(v)-q(v) +\sqrt{4b_0(v)p(v)+(p(v)+q(v)-s(v))^2}. \end{aligned}$$

Using methodology from Sect. 2, we construct an approximation of *Y* in the vicinity of the optimal strategy $v^*$. The coefficients obtained based on the approximation are then compared to the Taylor expansion of the above analytical expression (see Table 1). Note that we re-scaled our approximation to have the same constant term as in the analytical expression and the same maximal value of fitness. From the table, one can see that our method provides a good approximation of *Y*.

**Table 1:** Comparing the Taylor expansion terms (up to the second order) of the analytical expression of fitness from Morozov and Kuzenkov (2016) with its numerical approximation based on the new computational method (Sect. 2). The model parameters are the same as those listed in Fig. 2 caption with $\phi _{1}=\phi _{2}=1$

Terms in approximation	Coefficients for exact fitness	Coefficients of approximated fitness
Constant term	0.8747	0.8747
$b_{0}$	0.0278	0.0230
*s*	$-$ 0.8343	$-$ 0.6880
*q*	$-$ 1.1657	$-$ 1.1904
*p*	33.8488	34.7378
$b_{0}^{2}$	$-$ 0.0002	$-$ 0.00004
$b_{0}*s$	$-$ 0.0023	$-$ 0.0182
$b_{0}*q$	0.0023	0.0008
$b_{0}*p$	0.5199	0.5926
$s^{2}$	0.2449	0.5156
$s*q$	$-$ 0.4899	$-$ 0.4909
$s*p$	$-$ 3.4120	$-$ 4.2630
$q^{2}$	0.2449	0.2553
$q*p$	3.4120	3.8865
$p^{2}$	$-$ 305.6119	$-$ 329.8416

Moreover, we found that both analytical and computational methods produce close optimal trajectories for adults and juveniles. For the given set of parameters, the difference in terms of the corresponding Fourier coefficients is less than 0.5% of the absolute values, which highlights the efficiency of the method.

**Fig. 2:**
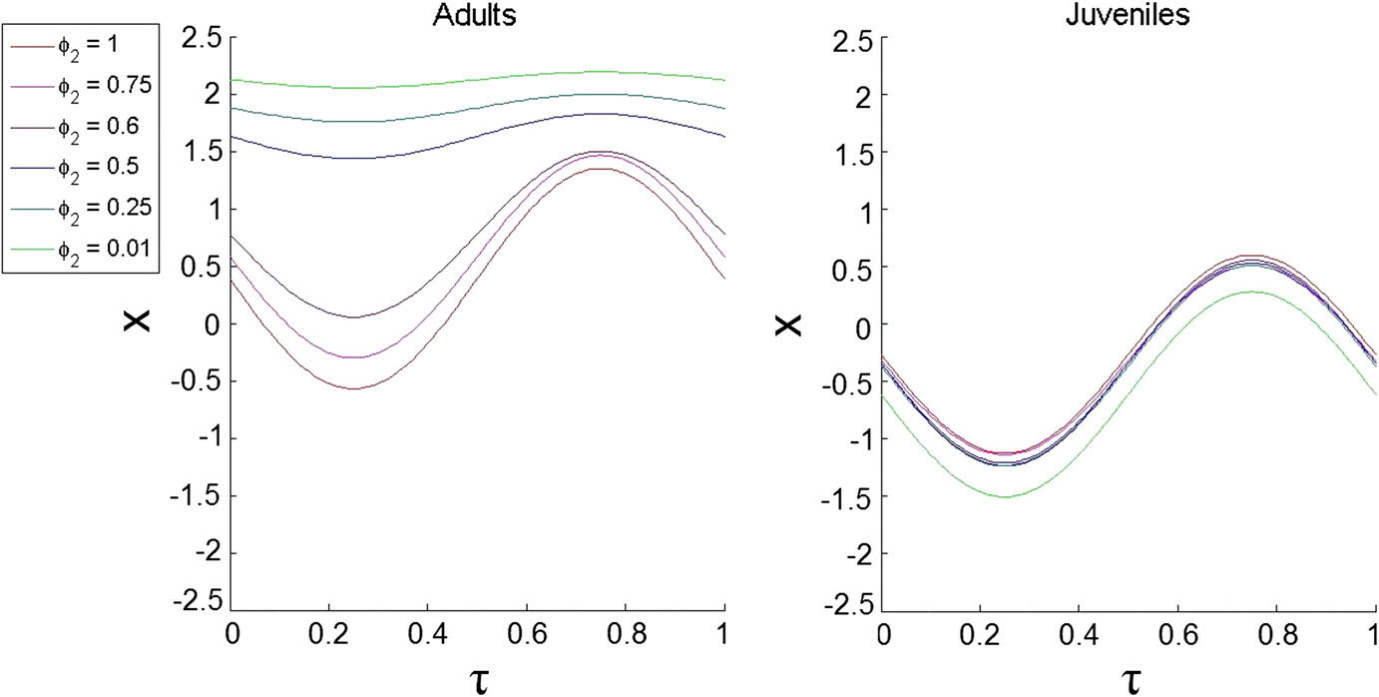
The dependence of the optimal trajectories of DVM on the competition coefficient $\phi _{2}$ under the static predation scenario ($F \equiv 0$). We considered the following values $\phi _{2}=0.01, \phi _{2}=0.25, \phi _{2}=0.5, \phi _{2}=0.6, \phi _{2}=0.75$ and $\phi _{2}=1$ for the model described by Eqs. (12), (13) and (14) with parameters $\phi _{1}=1, \epsilon _{A}=3, \xi =1, \sigma =1, \alpha _{A}=10, \alpha _{J}=1.1, \beta _{A}=0.05, \beta _{J}=\beta _{A}/5, \delta _{A}=0.1, \delta _{J}=\delta _{A}, \gamma _{A}=0.8, \gamma _{A}=\gamma _{J}$

Next, we consider a more complicated case of non-symmetric competition terms $\phi _{1} \ne \phi _{2}$. We explore the dependence of optimal DVM trajectories on the strength of competition between adults and juveniles described by varying $\phi _{2}$. Figure 2 shows how variation in $\phi _{2}$ would affect the amplitude and the average depth of vertical migration of both stages. For most trajectories, the model predicts night time feeding of zooplankton in the surface water and them staying in their refuge during the daytime. From the figure, one can see that an increase in $\phi _{2}$ results in a raise in the average depth of adults, whereas their amplitude decreases; for juveniles, the average depth decreases and the amplitude decreases only slightly. We can also see from the graphs that increasing $\phi _{2}$ has a greater effect on adults than juveniles; in fact, there is a sharp change in the behaviour of the adult zooplankton; they abruptly move from a medium average depth and amplitude to a very shallow average depth with a very small amplitude, i.e. DVM almost ceases. Thus, a non-symmetric increase in intraspecific competition of adult zooplankton reduces their DVM.

To investigate this jump further, we looked at how the main parameters are affected when we vary $\phi _{2}$, i.e. we explored the reproductive rate $b_0$ and total mortality rates of both adults and juveniles including the constant level of predation and the mortality due to intraspecific competition. We found that staying in surface waters by adults results in a large increase in $b_{0}$ which can compensate an increase in their mortality (graphs are not shown for the sake of brevity). Increasing the reproductive rate leads to a greater number of juveniles entering the system, which has a negative effect on the adults due to the intraspecific competition; however, it is compensated by a positive effect since more juveniles will eventually mature and become adults resulting in a greater number of reproducing adults. We conclude that overall it is advantageous for the adults to increase the reproductive rate and stay in surface waters despite an increase in predation.

**Fig. 3:**
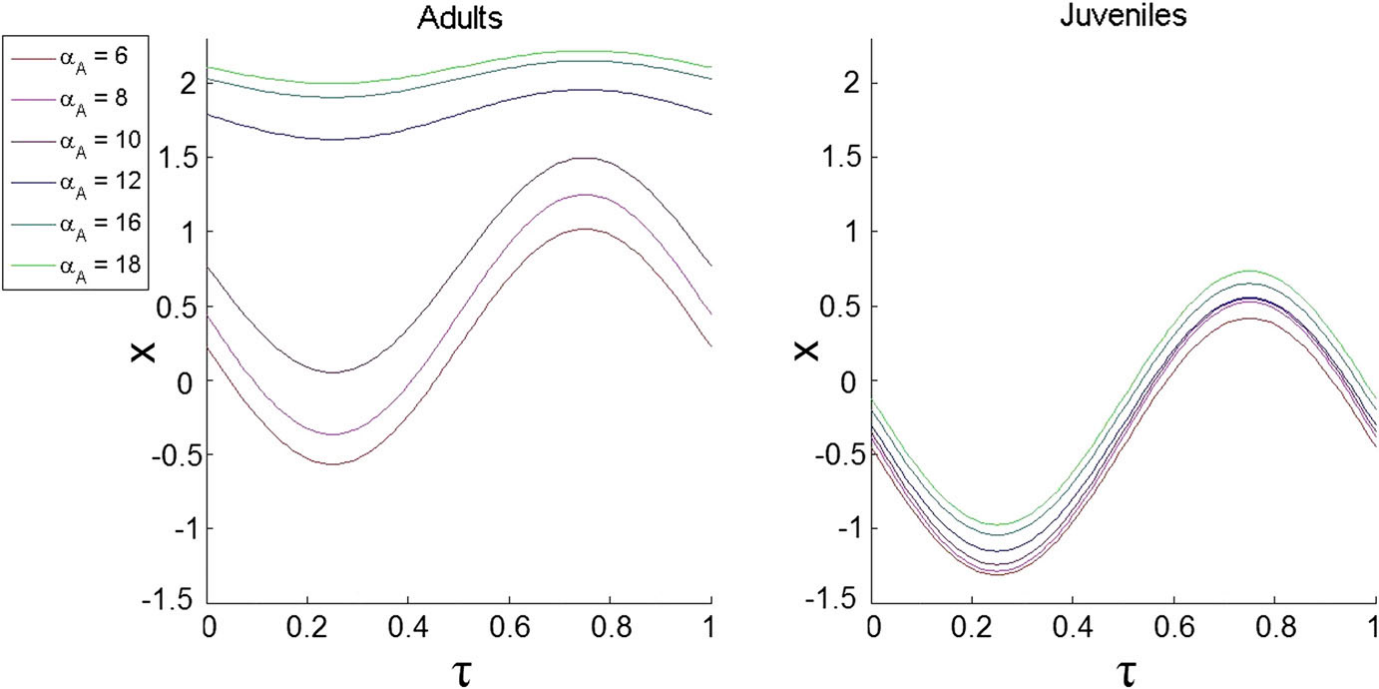
The dependence of the optimal trajectories of DVM on $\alpha _{A}$ under the static predation scenario ($F \equiv 0$). We considered the following values $\alpha _{A}=6, \alpha _{A}=8, \alpha _{A}=10, \alpha _{A}=12, \alpha _{A}=16$ and $\alpha _{A}=18$. The other parameters are $\phi _{1}=1, \phi _{2}=0.6, \epsilon _{A}=3, \xi =1, \sigma =1, \alpha _{J}=1.1, \beta _{A}=0.05, \beta _{J}=\beta _{A}/5, \delta _{A}=0.1, \delta _{J}=\delta _{A}, \gamma _{A}=0.8, \gamma _{A}=\gamma _{J}$

Variation of the total food level available for zooplankton results in major changes in patterns of optimal DVM. To demonstrate the dependence of trajectories on the availability of food, we independently varied $\alpha _{A}$ and $\alpha _{J}$. The resultant optimal trajectories for an increase in $\alpha _{A}$ are shown in Fig. 3. From the figure, one can see that initially increasing $\alpha _{A}$ causes the adults subpopulation to move to shallower waters, but at some point between $\alpha _{A}=10$ and $\alpha _{A}=12$ there is a drastic jump in behaviour as it is more advantageous for the adult zooplankton to stay in very shallow water and migrate very little. The observed jump is actually a result of coexistence of two points of maxima of *Y*: initially, the maximum corresponding to pronounced migration has a higher value of *Y*. A progressive increase of food density makes another peak of *Y* emerge, and with high $\alpha _{A}$ this peak eventually becomes dominant. Increasing $\alpha _{A}$ further, we observe that adults move to even shallower waters and reduce their amplitude even further, and DMV cases. On the contrary, for juveniles, increasing $\alpha _{A}$ causes their average depth to very slightly increase and only slightly decrease their amplitude of migration. Variation of $\alpha _{J}$ produces a similar effect. (The graphs are not shown for brevity).

### DVM with Dynamic Predation

Including dynamic predators may largely alter patterns of DVM. It is important to stress that Assumptions 1 and 2 are not always satisfied in this system and fitness may depend on initial conditions. However, by using similar reasoning as in Sect. 2.3 we can show that the numerical method can be still applied in these cases as well. In particular, the domains of feasible (allowed) parameters are strictly convex in the subspaces of parameters describing life history traits of juveniles and adults, where fitness function becomes linear (see SM4). An estimate of the number of points required for Step 1 gives 300 points (calculated in a similar way as in SM2).

Here, we mostly focused on the realistic case where the predator consumes both adult and juvenile zooplankton, with a smaller level of predation on juveniles than adults, so $\gamma _{F_{A}},\gamma _{F_{J}}\ne 0$ with $\gamma _{F_{A}}>\gamma _{F_{J}}$. Our simulations, however, show that in other cases (e.g. $\gamma _{F_{A}}=0$ or $\gamma _{F_{J}}=0$) optimal patterns of DVM behave in a similar way.

**Fig. 4:**
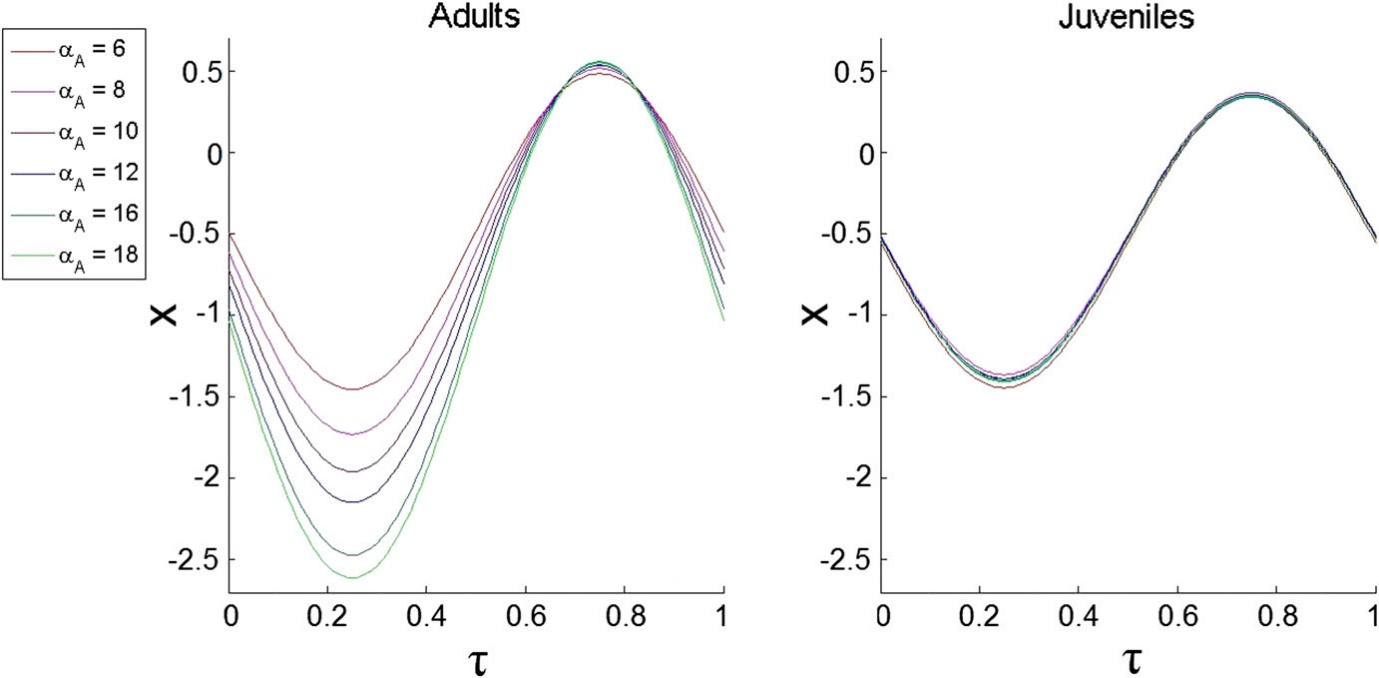
The dependence of the optimal trajectories on the available food for adults $\alpha _{A}$ under the dynamic predator scenario. We consider the following values $\alpha _{A}=6, \alpha _{A}=8, \alpha _{A}=10, \alpha _{A}=12, \alpha _{A}=16$ and $\alpha _{A}=18$. The other parameters are $e=0.5, m_{F}=0.01, \gamma _{F_{A}}=0, \gamma _{F_{J}}=0.5, \phi _{1}=0.5, \phi _{2}=\phi _{1}, \epsilon _{A}=3, \xi =1, \sigma =1, \alpha _{J}=1.1, \beta _{A}=0.05, \beta _{J}=\beta _{A}/5, \delta _{A}=0.1, \delta _{J}=\delta _{A}, \gamma _{A}=0.8$ and $\gamma _{A}=\gamma _{J}$

We explore the dependence of DVM on the amount of food available for adults described by $\alpha _{A}$ to compare the results to the case of static predators. The optimal patterns of DVM are plotted in Fig. 4. From the figure, one can see that by increasing the level of food for zooplankton $\alpha _{A}$ has only a small impact on the DVM of juveniles. However, the trajectory for adults is strongly affected by $\alpha _{A}$. Indeed, increasing $\alpha _{A}$ decreases the average depth and enlarges the amplitude of DVM to ensure that during dark hours when visual predators are not effective, the adults stay in surface waters to consume enough phytoplankton and therefore have enough energy to migrate and reproduce. Interestingly, with the addition of a dynamic predator, we no longer observe the jump in behaviour as in Fig. 3. Thus, the positive effect of consumption of a large amount of phytoplankton (cf. Fig. 3) by adults staying in shallow waters would be negligible as compared to a disproportional increase in predation pressure: the fish density *F* will increase with the overall amount of phytoplankton. These conclusions are also supported by numerically plotting optimal values of the reproductive and the mortality rates for both adults and juveniles for increasing $\alpha _{A}$. (The results are not shown here for the sake of brevity.)

Finally, we checked the influence of a gradual increase of $\alpha _A$ on patterns of optimal DVM in the case where the dynamic predator only consumes one zooplankton stage: juveniles or adults. Surprisingly, we found very similar patterns of the dependence of DVM on $\alpha _A$ as those shown in Fig. 4, i.e. when *F* can feed on both *A* and *J*. We do not show the results for the sake of brevity.

## Discussion and Conclusions

In this paper, we introduce a new method of computation of the evolutionarily optimal life history traits and behavioural patterns which is based on the approximation of the fitness function(al). Defined in Sect. 2, fitness describes the mutual hierarchy of competitive advantage orders of organisms’ strategies. This idea is close to the well-known generic concept of fitness in evolutionary biology: it is often assumed that natural selection should result in an increase of the organism’s fitness (this is known as a ‘hill-climbing’ process); the evolutionarily optimal behaviour would correspond to a maximum of the fitness function subject to some trade-offs (Wright 1932; Roff 1992; Davies et al. 2012; Birch 2016). The new method allows us to reconstruct fitness directly from underlying population dynamics equations based on the comparison of the ranking order of a relatively small number of strategies. (Of course, this number would depend on the complexity of the model.) Among the advantages of our method is the possibility to find optimal strategies for function-valued traits (see SM3). Moreover, by considering a large number of points in Step 1 we can make sure to reach the global maximum of fitness function thus avoiding the situation of getting stuck at some local maximum. Overall, the idea of our method is somewhat close to the optimisation principle in adaptive dynamics using maximisation of the invasion fitness of a mutant into a resident population (Gyllenberg and Service 2011; Gyllenberg et al. 2011). However, the formal correspondence between our method and the one in adaptive dynamics still needs to be established. For example, in the adaptive dynamics framework, evolutionary outcomes can be calculated through pure optimisation only when there is but a single feedback variable.

Among the possible advantages of our method are the following. This method seems to be efficient in the case where the strategy *v* is a function-valued trait and it is expected to have a complicated shape including several maxima/minima or some points of discontinuity. Also, the method seems to be practical in the situation with population structuring: in this case, *v* becomes a vector of functions. For example, it is well known that marine herbivorous zooplankton often includes up to 6 developmental stages (Huntley and Brooks 1982). In the above-mentioned situation, one needs to use an analytical or semi-analytical technique to reconstruct functions included in the vector *v*, for example, using calculus of variations or optimal control. Our method allows the implementation of such semi-analytical tools using the explicit approximation of the fitness functional (see SM3). Implementation of other approaches, for example, adaptive dynamics may be less efficient. For example, deriving the invasion fitness in adaptive dynamics for a population model with a large number of developmental stages usually gives an implicit expression which one should use to further derive Euler equations. Although in principle we are still able to write cumbersome expressions for the optimal strategies, they will be most likely analytically intractable and are hard to be solved numerically. Note that in the case of the population dynamics attractors are non-stationary (e.g. limit cycles or chaotic attractors), we are generally unable to apply analytical techniques, and our method of numerical reconstruction of fitness might be useful in this case. We should stress that our method is designed to work along with the existing methods of search for optimal strategies rather than to replace them.

Interestingly, our numerical method can be used in the case where we do not know the exact underlying model equations, for example, when we have empirical times series of long-term population dynamics of similar competing species. In this case, we assume that the underlying system is such that we can construct a fitness function (this fact, however, can be hard to verify for an insufficient amount of data). To apply the method, we need to first choose the key characteristics of species (e.g. foraging rate, mortality, maturation time, etc.). Further, one needs to go through step 2 in Sect. 2, by using data-based estimates of growth rates and re-ordering species according to the growth rates. Realistically, based on a limited number of species, we should expect to use a linear rather than a quadratic function to approximate the generalised fitness. Finally, using the obtained fitness function it can be possible to predict the fate of a new species with known characteristics. Note that the same idea can be implemented in economics and business modelling when deciding whether or not a new product will be competitive with respect to the existing products based on the known dynamics on sales numbers and products’ characteristics. The ranking order can be derived based on the comparison of the dynamics of sales rates.

We should admit that the method itself has some restrictions to its applicability. In particular, it is mainly applicable to models where the ranking order does not depend on initial conditions and their fitness is frequency independent. The class of such models can be narrow (as compared to all possible population models in mathematical biology). However, this class does include a number of biologically meaningful models as a single-species population model with age structure [Model (12)–(13) with a static predator], models with continuous age of von-Foerster equation type (Kuzenkov and Morozov 2019) or a Lotka–Volterra predator–prey model (Morozov and Kuzenkov 2016). Note that verification of the assumptions in Sect. 2 can be done numerically by running simulations with different initial conditions; however, in practice this can require running a large number of simulations. Interestingly, the method can be still implemented to some models which have formally frequency-dependent fitness (i.e. their fitness depends on initial conditions). An example of such a situation is given in Sects. 2.3 and 4.2, where fitness can be well approximated in the vicinity of its maximum. Note that at the moment, the limits of applicability of the method are an open question. In particular, the possibility of its implementation for a frequency-dependent fitness may depend on the shape of the domain of viable parameters; for example, in Sects. 2.3 and 4.2 we suggest that parametric domain is strictly convex. We admit that further research is needed to explore the limits of applicability of the method to models where fitness depends on initial conditions.

In the initial framework, we assumed the system to have a strong inheritance with no mutations. In this case, all possible strategies in the system will be those with nonzero initial conditions $\rho (v,0)>0$. We can easily extend this framework by allowing the introduction of mutants with any strategy (which should still respect our trade-offs) which can also be considered as an invasion of the system by similar subspecies from other ecosystems. In the case where rates of introduction of mutants are low, the resultant evolutionary outcome will always be the same as in the system with a strong inheritance: the surviving strategies will be the ones which maximise fitness.

As the study case, we implemented our technique to reveal the optimal patterns of DVM of zooplankton in oceans and lakes using a population model of stage-structured zooplankton with a dynamic visual predator, i.e. when the biomass of the predator is determined by the strategy that its prey (zooplankton) uses. The previous models of DVM only considered a static predator and constant environment (see references in Sect. 3.1 for details). Our findings show that considering static and dynamic predator scenarios can result in completely different conclusions in terms of the amplitude of DVM (cf. Figs. 3, 4). The fact that zooplankton intensifies DVM in a food-rich environment in the system with a dynamic predator, whereas animals stop migrating in the case of static predators can be easily explained. In the case of the dynamic predator, an increase in food for zooplankton results in an increase in the population size of predators consuming them. Thus, staying and feeding by zooplankton in surface waters would result in a further increase of the numbers of predators which would not compensate for the increase in zooplankton reproduction rate. In this case, the best strategy will be staying away from the surface as much as possible (see Fig. 4). For the static predator, the staying and grazing of zooplankton in surface food-rich waters would compensate for their high mortality in the absence of DVM (see Fig. 3). Interestingly, empirical observation of DVM in a food-rich environment for zooplankton grazers is contradictory: some studies point to a reduction in the amplitude of migration in the case that food is abundant (Gliwicz 1986; Gabriel and Thomas 1988), whereas others point out the opposite behaviour, i.e. a pronounced increase in DVM under food-rich conditions (Huntley and Brooks 1982). Using our modelling results, we can hypothesise that the difference in responses of DVM to the food abundance may be explained due to the long-term prevalence of a particular type of predation: dynamic or static in the considered ecosystems in the past. Another new finding in DVM is an abrupt switch between migrating and non-migrating strategies (Figs. 2, 3) which was not reported in previous models. We can explain this phenomenon as a result of the occurrence of several maxima of fitness in the space of strategies and the switching between them when an external parameter is gradually changed.

Note that we explored DVM using a simple two-stage model of zooplankton dynamics as an illustrative example. The model coefficients specified in Sect. 3.3 were obtained based on simple parameterisations from Morozov and Kuzenkov (2016). Our framework allows us to include more developmental stages and to incorporate more realistic functions for vertical profiles of phytoplankton, temperature, oxygen and salinity as well as more accurate descriptions of daily predator activity (Mangel and Clark 1988). Also, one can consider saturation in the functional response of the visual predators; this can cause oscillations of species densities as in the classical Rosenzweig–MacArthur model (Roy and Chattopadhyay 2007), thus the ordering of species in terms of their per capita growth rates given by (3) should be done via averaging over the period of oscillations. This would be an interesting extension of the current work.

## Electronic supplementary material

Below is the link to the electronic supplementary material.

Supplementary material 1 (docx 204 KB)

## References

[CR1] Birch J (2016). Natural selection and the maximization of fitness. Biolo Rev.

[CR2] Bianchi D (2007). Intensification of open-ocean oxygen depletion by vertically migrating animals. Nat Geosci.

[CR3] Broom M, Rychtar J (2013). Game-theoretical models in biology.

[CR4] Buesseler K, Lamborg C, Boyd P (2007). Revisiting carbon flux through the oceans twilight zone. Science.

[CR5] De Robertis A (2002). Size-dependent visual predation risk and the timing of vertical migration: an optimization model. Limnol Oceanogr.

[CR6] Davies NB, Krebs JR, West SA (2012). An introduction to behavioural ecology.

[CR7] Ducklow H, Steinberg D, Buesseler K (2001). Upper ocean carbon export and the biological pump. Oceanography.

[CR8] Fiksen O, Giske J (1995). Vertical distribution and population dynamics of copepods by dynamic optimization. ICESJ Mar Sci.

[CR9] Fiksen O, Carlotti F (1998). A model of optimal life history and diel vertical migration in *Calanus finmarchicus*. Sarsia.

[CR10] Fortier M, Fortier L, Hattori H, Saito H, Legendre L (2001). Visual predators and the diel vertical migration of copepods under arctic sea ice during the midnight sun. J Plankton Res.

[CR11] Gabriel W, Thomas B (1988). Vertical migration of zooplankton as an evolutionarily stable strategy. Am Nat.

[CR12] Gyllenberg M, Service R (2011). Necessary and sufficient conditions for the existence of an optimisation principle in evolution. J Math Biol.

[CR13] Gyllenberg Mats, Metz J. A. J. Hans, Service Robert (2011). When Do Optimisation Arguments Make Evolutionary Sense?. The Mathematics of Darwin’s Legacy.

[CR14] Geller W (1986). Diurnal vertical migration of zooplankton in a temperate great lake (L.Constance): a starvation avoidance mechanism?. Arch Hydrobiol Suppl.

[CR15] Geritz SAH, Kisdi E, Meszena G, Metz JAJ (1998). Evolutionarily singular strategies and the adaptive growth and branching of the evolutionary tree. Evol Ecol.

[CR16] Gliwicz ZM (1986). A lunar cycle in zooplankton. Ecology.

[CR17] Gilliam J, Fraser D (1987). Habitat selection under predation hazard: test of a model with foraging minnows. Ecology.

[CR18] Gorban AN (2007). Selection theorem for systems with inheritance. Math Model Nat Phenom.

[CR19] Hamblin S (2013). On the practical usage of genetic algorithms in ecology and evolution. Methods Ecol Evol.

[CR20] Han BP, Straskraba M (1998). Modelling patterns of zooplankton diel vertical migration. J Plankton Res.

[CR21] Han BP, Straskraba M (2001). Control mechanisms of diel vertical migration: theoretical assumptions. J Theor Biol.

[CR22] Hansen AN, Visser AW (2016). Carbon export by vertically migrating zooplankton: an optimal behavior model. Limnol Oceanogr.

[CR23] Hays G (2003). A review of the adaptive significance and ecosystem consequences of zooplankton diel vertical migrations. Hydrobiologia.

[CR24] Hellweger FL, Clegg RJ, Clark JR, Plugge CM, Kreft JU (2016). Advancing microbial sciences by individual-based modelling. Nat Rev Microbiol.

[CR25] Hofbauer J, Sigmund K (1998). Evolutionary games and population dynamics.

[CR26] Horst R, Pardalos PM, Thoai NV (1995). Introduction to global optimization.

[CR27] Huntley M, Brooks ER (1982). Effects of age and food availability on diel vertical migration of *Calanus pacificus*. Mar Biol.

[CR28] Iwasa Y (1982). Vertical migration of zooplankton: a game between predator and prey. Am Nat.

[CR29] Kaiser MJ (2005). Marine ecology: processes, symptoms and impacts.

[CR30] Kisdi E, Geritz SAH (2010). Adaptive dynamics: a framework to model evolution in the ecological theatre. J Math Biol.

[CR31] Kisdi E, Priklopil T (2011). Evolutionary branching of a magic trait. J Math Biol.

[CR32] Krantz D, Luce RD, Suppes P, Tversky A (1971). Foundations of measurement: additive and polynomial representations.

[CR33] Kuzenkov OA, Kuzenkova GV (2012). Optimal control of self-reproducing systems. J Comput Syst Sci Int.

[CR34] Kuzenkov OA, Ryabova EA (2015). Variational principle for self-replicating systems. Math Model Nat Phenom.

[CR35] Kuzenkov OA, Ryabova EA (2015). Limit possibilities of solution of a hereditary control system. Differ Equ.

[CR36] Kuzenkov OA, Morozov A (2019) Towards constructing a mathematically rigorous framework for modelling evolutionary fitness. Bull Math Biol. 10.1007/s11538-019-00602-3 10.1007/s11538-019-00602-3 PMC6874698 30949887

[CR37] Lampert W (1989). The adaptive significance of diel vertical migration of zooplankton. Funct Ecol.

[CR38] Levich AP (2000). Variational modelling theorems and algocoenoses functioning principles. Ecol Model.

[CR39] Liu SH, Sun S, Han BP (2003). Diel vertical migration of zooplankton following optimal food intake under predation. J Plankton Res.

[CR40] Liu SH, Sun S, Han BP (2006). Viewing DVM via general behaviors of zooplankton: away bridging the success of individual and population. J Theor Biol.

[CR41] Mangel M, Clark CW (1988). Dynamic modelling in behavioural ecology.

[CR42] McLaren I (1963). Effects of temperature on growth of zooplankton, and the adaptive value of vertical migration. J Fish Board Can.

[CR43] McNamara JM, Houston AI, Collins EJ (2001). Optimality models in behavioral biology. SIAM Rev.

[CR44] Morozov A, Best A (2012). Predation on infected host promotes evolutionary branching of virulence and pathogens’ biodiversity. J Theor Biol.

[CR45] Morozov AY, Kuzenkov OA (2016). Towards developing a general framework for modelling vertical migration in zooplankton. J Theor Biol.

[CR46] Ohman M (1991). The demographic benefits of diel vertical migration by zooplankton. Ecol Monogr.

[CR47] Parvinen K, Dieckmann U, Heino M (2006). Function-valued adaptive dynamics and the calculus of variations. J Math Biol.

[CR48] Pearre S (2003). Eat and run? The hunger/satiation hypothesis in vertical migration: history, evidence and consequences. Biol Rev.

[CR49] Ringelberg J (2003). Diel vertical migration of zooplankton in lakes and oceans.

[CR50] Roff DA (1992). The evolution of life histories: theory and analysis.

[CR51] Roy S, Chattopadhyay J (2007). The stability of ecosystems: a brief overview of the paradox of enrichment. J Biosci.

[CR52] Sainmont J, Andersen KH, Thygesen UH, Fiksen O, Visser AW (2015). An effective algorithm for approximating adaptive behavior in seasonal environments. Ecol Model.

[CR53] Sumida BH, Houston AI, McNamara JM, Hamilton WD (1990). Genetic algorithms and evolution. J Theor Biol.

[CR54] Wright S (1932) The roles of mutation, inbreeding, crossbreeding, and selection in evolution. In: Proceedings of the sixth international congress of genetics, vol 1. Brooklyn Botanic Garden

[CR55] Zhigljavsky AA, Zilinskas A (2008). Stochastic global optimization.

