# Supplementary material for: Revealing Evolutionarily Optimal Strategies in Self-Reproducing Systems via a New Computational Approach
Source: Bull Math Biol. 2019 Nov 18;81(11):4701–25. doi: 10.1007/s11538-019-00663-4 (PMC6874526; doi:10.1007/s11538-019-00663-4)
Supplement: Supplementary file 1 — Supplementary material 1 (docx 204 KB) [file 11538_2019_663_MOESM1_ESM.docx]

**Supplementary material for ‘Revealing Evolutionarily Optimal Strategies in Self-Reproducing Systems via a New Computational Approach’ by Sandhu et al., 2019**

**SM1**

Here prove **Proposition 1** stated in the main text.

Let (*c^*^*, *r^*^*) be the best point (corresponding to the best strategy of prey in model (8)-(9)) in the set *Q* of parameters (*c*, *r*). For this point we have

$Y_{1}\left( c*,r*, c*,r* \right)={max}_{Q} Y_{1}\left( c,r, c*,r* \right)$,

where the fitness function *Y_1_* is defined by (13).

For an arbitrary ε>0 we consider the set $I\subset Q$ such that for its points we have

$Y_{1}\left( c,r, c*,r* \right)>Y_{1}\left( c*,r*, c*,r* \right)-\varepsilon$*.*

Any point of *I* can be regarded as an *ε*-approximation of the best point (*c^*^*, *r^*^*) according to the value of *Y_1_*. Since $Y_{1}\left( c,r, c*,r* \right)$ is assumed to be uniformly continuous in *Q*, there exists a neighbourhood of the point (*c^*^*, *r^*^*) in $I\subset Q$. Hence, the set *I* has a non-zero measure (the area). The (relative) complement set *Q\I* should be understood as the set of “*ε*-unfavourable” points. The approximate numerical optimisation of $Y_{1}\left( c,r, c*,r* \right)$ in *Q* will consist in finding at least one point from *I*. In other words, the optimisation problem will be solved up to an accuracy ε in terms of the value of fitness *Y_1_*.

Since the function *Y_1_* is uniformly continuous in *Q^2^* as a function of all four variables, for the given *ε*>0 there exist a *δ*>0 such that the following inequality holds

$$|Y_{1}\left( с_{1},r_{1}, c_{2},r_{2} \right)-Y_{1}\left( с_{3},r_{3}, c_{4},r_{4} \right)|<\varepsilon/4$$

as soon as the distance between $\left( с_{1},r_{1}, c_{2},r_{2} \right)$ and $\left( с_{3},r_{3}, c_{4},r_{4} \right)$ becomes smaller than *δ*.

In this case, for any point (*c', r'*) located in the vicinity of the *δ*-neighbourhood of (*c^*^, r^*^*) the following inequalities will hold

$$Y_{1}\left( c*,r*, c*,r* \right)-Y_{1}\left( c^{'},r', c*,r* \right)<\varepsilon/4$$

$$|Y_{1}\left( c,r, c*,r* \right)-Y_{1}\left( {c,r,c}^{'},r' \right)|<\varepsilon/4$$

for any point $\left( c,r \right)$ from *Q.* In other words,

$$Y_{1}\left( c^{'},r', c*,r* \right)>Y_{1}\left( c*,r*, c*,r* \right)-\varepsilon/4$$

$$Y_{1}\left( c,r, c*,r* \right)-\frac{\varepsilon}{4}<Y_{1}\left( {c,r,c}^{'},r^{'} \right)<Y_{1}\left( c,r, c*,r* \right)+\varepsilon/4$$

for any point $\left( c,r \right)$ from *Q.*

If for $\left( c,r \right)$ we have

$$Y_{1}\left( c,r, c*,r* \right)<Y_{1}\left( c*,r*, c*,r* \right)-3\varepsilon/4$$

(in particular, we may consider $(c,r)\in Q/I$), we then obtain

$$Y_{1}\left( {c,r,c}^{'},r' \right)<Y_{1}\left( c,r, c*,r* \right)+\frac{\varepsilon}{4}<Y_{1}\left( c*,r*, c*,r* \right)-\frac{3\varepsilon}{4}+\frac{\varepsilon}{4}=Y_{1}\left( c*,r*, c*,r* \right)-\frac{\varepsilon}{2}$$

$Y_{1}\left( {c^{'},r',c}^{'},r' \right)>Y_{1}\left( c^{'},r', c*,r* \right)-\varepsilon/4>Y_{1}\left( c*,r*, c*,r* \right)-\varepsilon/2$*.*

In other words,

$Y_{1}\left( {c,r,c}^{'},r' \right)<Y_{1}\left( {c^{'},r',c}^{'},r' \right)$.

Hence, if a certain set $Q_{0}\subset Q$ contains at least one point from the *δ*-neighbourhood of (*c^*^, r^*^*), then the best point of $Q_{0}$ cannot belong to *Q\I* (i.e. to the set of unfavourable points).

Note that in general in a randomly generated sequence of points (that we use to find an approximate solution to the optimisation problem) there can be a pair of points (*c_1_,r_1_*) and (*c_2_,r_2_*) that outcompetes all other points, including ${(c}^{'},r')$. In this case, at least for one point from the above pair, for example, for (*c_1_,r_1_*) we should have

$Y_{1}\left( c_{1},r_{1}, c^{'},r' \right)>Y_{1}\left( {c^{'},r',c}^{'},r' \right)$,

otherwise ${(c}^{'},r')$ should outcompete both (*c_1_,r_1_*) and (*c_2_,r_2_*). Hence, we have

$Y_{1}\left( c_{1},r_{1}, c*,r* \right)> Y_{1}\left( c_{1},r_{1}, c^{'},r' \right)-\varepsilon/4>Y_{1}\left( c*,r*, c*,r* \right)-3\varepsilon/4$*.*

However, this signifies that (*c_1_*, *r_1_*) cannot be located in *Q\I*. Consequently, in the case of the coexistence of two best points in the generated sequence, at least one point of this pair should belong to the set *I*, i.e. to be an *ε*-approximation of (*c^*^*, *r^*^*).

In conclusion, by generating a large number of points using some infinite (random) sequence which is everywhere dense in *Q*, with the probability of 1 we will have a point landed in *I* (for any *ε>*0). This will provide an *ε*-approximation of the fitness function. An estimate for the required average number of points in the Monte-Carlo algorithm is further provided in SM2.

**SM2**

The critical number of points *N_cr_* required in Step 1 when implementing the Monte-Carlo method for model (8)-(9) can be estimated in the following way. We fix a positive number *δ* which defines the closeness (or the distance) of a given point to the best point (*c^*^*, *r^*^*) in domain *Q*. We require that at least one point out of generated sequences of points should land in the *δ*-neighbourhood of (*c^*^*, *r^*^*).

Let us assume that *Q* is a bounded set, i.e. $0<c_{min}\leq c\leq c_{max}$; $0<r_{min}\leq r\leq r_{max}$. In the case where the distance between $\left( с_{1},r_{1}, c_{2},r_{2} \right)$ and $\left( с_{3},r_{3}, c_{4},r_{4} \right)$ becomes smaller than *δ*, we have

$$\left| Y_{1}\left( с_{1},r_{1}, c_{2},r_{2} \right)-Y_{1}\left( с_{3},r_{3}, c_{4},r_{4} \right) \right|=\left| r_{1}-с_{1}\left( \frac{r_{2}}{c_{2}}-\frac{1}{{c_{2}}^{2}} \right)-r_{3}+с_{3}\left( \frac{r_{4}}{c_{4}}-\frac{1}{{c_{4}}^{2}} \right) \right|\leq\left| r_{1}-r_{3} \right|+\left| {(с}_{3}-с_{1})\left( \frac{r_{4}}{c_{4}}-\frac{1}{{c_{4}}^{2}} \right) \right|+с_{1}\left| \frac{r_{4}}{c_{4}}-\frac{1}{{c_{4}}^{2}}-\frac{r_{2}}{c_{2}}+\frac{1}{{c_{2}}^{2}} \right|\leq\left| r_{1}-r_{3} \right|+\left| с_{3}-с_{1} \right|\left( \frac{r_{max}}{c_{min}}+\frac{1}{{c_{min}}^{2}} \right)+c_{max}\left| \frac{{c_{2}r}_{4}-{c_{4}r}_{2}}{c_{4}c_{2}} \right|+c_{max}\left| \frac{{c_{2}}^{2}-{c_{4}}^{2}}{{c_{4}}^{2}{c_{2}}^{2}} \right|\leq\left| r_{1}-r_{3} \right|+\left| с_{3}-с_{1} \right|\left( \frac{r_{max}}{c_{min}}+\frac{1}{{c_{min}}^{2}} \right)+c_{max}\frac{\left| r_{4}-r_{2} \right|}{c_{min}}+\frac{c_{max}r_{max}}{c_{min}^{2}}\left| c_{2}-c_{4} \right|+\frac{2c_{max}^{2}}{c_{min}^{4}}\left| c_{2}-c_{4} \right|<\left( 1+\left( \frac{r_{max}}{c_{min}}+\frac{1}{{c_{min}}^{2}} \right)+\frac{c_{max}}{c_{min}}+\frac{c_{max}r_{max}}{c_{min}^{2}}+\frac{2c_{max}^{2}}{c_{min}^{4}} \right)\delta\equiv\Delta\delta.$$

Thus if *δ*<*ε*/4*Δ*, then the above inequality holds.

The probability of lending of a point in the *δ*-neighbourhood of (*c^*^*, *r^*^*) is given by *μ*(*O_δ_*)/*μ*(*Q*), where *μ*(*O_δ_*) is the measure (the area) of the *δ*- neighbourhood, *μ*(*Q*) is the measure (the area) of the whole available parameter space. Here we suggest that of simplicity we generate a uniformly random sequence of points. The probability of missing the *δ*-neighbourhood of (*c^*^*, *r^*^*) by a point is given by 1− *μ*(*O_δ_*)/*μ*(*Q*). For *N* points, the probability of missing the *δ*-neighbourhood is given by (1− *μ*(*O_δ_*)/*μ*(*Q*))*^N^*, and the probability of lending of at least one point out of *N* is given by 1− (1− *μ*(*O_δ_*)/*μ*(*Q*))*^N^*. We can set the probability of getting to the *δ*-neighbourhood to be 1− *θ*, where *θ* is a positive constant. The required average number of points *N_cr_* will be determined by the inequality

1− (1− *μ*(*O_δ_*)/*μ*(*Q*))*^Ncr^* > 1− *θ*  or *N_cr_* >log _1- μ(Oδ)/μ(Q)_ *θ*.

For the example based on Model (8)-(9), we can estimate the critical number of points as considering *ε*=0.02 and some arbitrary parameters *r_max_* =5; *c_min_* =1; *c_max_* =7 we obtain *Δ*$\approx$150 and the critical can be estimated as *δ*$\approx1.8$. Further, we consider that *μ*(*O_δ_*)/*μ*(*Q*)$\approx(\frac{\delta}{c_{max}-c_{min}})(\frac{\delta}{r_{max}})$ = 0.108. From this, the critical number of points will be given (1− *θ* =0.99) as *N_cr_* >50 points.

Note that in some cases we can estimate the size of *δ*-neighbourhood from some biological rationale, for example in the case of DVM of zooplankton, one can take *δ* as 1-2m, which is usually up to 5% of the total euphotic zone of the water column.

**SM3**

Here we derive the optimal strategy of DVM from the known generalised fitness function *Y* using analytical methods of calculus of variations.

For brevity, we consider here the population model from Section 3.2 of the main text in the absence of fish, i.e. *F* ≡0. The case with *F*(*t*) >0 can be addressed similarly. We re-write the model coefficients introduced in Section 3.3 as (for simplicity, here we use *t* as the integration variable, which should not (probably) cause confusion)

(reproduction coefficient of adults),

$s\left(x_{A}\right)=\int_{0}^{1} G_{s}\left(x_{A}\right)dt$ (mortality rate of adults),

$p\left(x_{J}\right)=\frac{\eta L\left(x_{J}\right)}{L\left(x_{J}\right)+\kappa}$ (transition rate from juveniles to adults) with

(energy gain of juveniles),

$q\left(x_{J}\right)=\int_{0}^{1} G_{q}\left(x_{J}\right)dt$ (mortality rate of juveniles).

Here *x_A_*, *x_J_* are the strategies of DVM of adults and juveniles, respectively. The functions inside the above integrals can be easily identified through comparison to those in Section 3.3

Due to the daily periodicity of migrations we have *x_A_*(0) = *x_A_*(1), *x_J_*(0) = *x_J_*(1).

The fitness function is given by the following quadratic form as function of *b_0_*, *s*, *p*, *q* (here for simplicity we use coefficients α_i_, α_ij_)

*Y*=*α_1_b_0_+α_2_s+α_3_p+α_4_q+α_11_b_0_^2^+ α_12_b_0_s+α_13_b_0_p+α_14_b_0_q+α_22_s^2^+α_23_sp+α_24_sq+α_33_p^2^+α_34_pq+α_44_q^2^.*

We need to find *x_A_*, *x_J_* which maximise the above functional.

We will derive the equations of the necessary condition of the extremum of *Y*.

Let us denote $x'_{J}=u_{J}$; $x'_{A}=u_{A}$. We introduce some arbitrary continuous functions *h*(*t*) and *g*(*t*) t$\in$[0,1]. We consider the variation of the functions defined as (*ε* is a parameter)

*u_Jε_*(*t*)=*u_J_* (*t*) + *εh*(*t*), *u_Aε_*(*t*)=*u_A_*(*t*) + *εg*(*t*).

Then we have

Δ*u_J_*(*t*)=*u_J_*(*t*) + *εh*(*t*) − *u_J_*(*t*)=*εh*(*t*), Δ*u_A_*(*t*)=*u_A_*(*t*) + *εg*(*t*) − *u_A_*(*t*)=*εg*(*t*),

$\Delta x'_{J}=\Delta u_{J}=\varepsilon h(t)$; $\Delta x'_{A}=\Delta u_{A}=\varepsilon g(t)$.

The variation derivative is given by

*dY*/*dε*=∂*Y*/∂*b* *db_0_*/*dε+∂Y*/*∂s ds*/*dε+∂Y*/*∂p dp*/*dε+∂Y*/*∂q dq*/*dε*=

=(*α_1_+ α_12_s^*^+ α_13_p^*^+α_14_q^*^+2α_11_ b_0_^*^*) *db*/*dε* +(*α_2_+ α_12_ b_0_^*^ +α_23_p^*^+α_24_q^*^ +2α_22_s^*^*) *ds*/*dε* +

(*α_3_+ α_13_ b_0_^*^+α_23_s^*^+α_34_q^*^+2α_33_p^*^*) *η κ* /(*L*(*p^*^*)+*κ*)^2^ *dL*/*dε* +(*α_4_+α_14_ b_0_^*^ +α_24_s^*^+α_34_p^*^+2α_44_q^*^*) *dq*/*dε*

Here *b_0_*^*^, *s*^*^, *p*^*^, *q*^*^ are parameters corresponding to the optimal strategies; *db_0_*/*dε*, *ds*/*dε*, *dL*/*dε*, *dq*/*dε* are the variation derivatives of the corresponding functionals.

We find the variation of the integral for *b_0_*

,

where *θ* is a number between zero and one (here we use the Lagrange theorem).

We introduce a function *ψ_Abε_* which satisfies the following adjoint equation

$$\psi'_{Ab\varepsilon}=-\partial G_{b1}\left(x_{A}+\theta x_{A}\right)/\partial x$$

and the boundary conditions *ψ_Abε_*(0)= *ψ_Abε_*(1).

Then we have

$\left(\psi_{Ab\varepsilon}\Delta x_{A}\right)^{'}=\psi_{Ab\varepsilon}\Delta u_{A}-\Delta x_{A}\partial G_{b1}\left(x_{A}+\theta x_{A}\right)/\partial x$.

Integration of the above equation gives

$\int_{0}^{1} \psi_{Ab\varepsilon}\Delta u_{A}dt=\int_{0}^{1} \Delta x_{A}\partial G_{b1}\left(x_{A}+\theta x_{A}\right)/\partial xdt$.

We obtain

$\frac{\Delta b_{0}}{\varepsilon}=\int_{0}^{1} \psi_{Ab\varepsilon}g+g \partial G_{b2}\left(u_{A}+\theta u_{A}\right)/\partial u dt$.

By taking the limit of the above expression ε→0 we obtain

$\frac{db_{0}}{d\varepsilon}=\int_{0}^{1} \psi_{Ab}g+g \partial G_{b2}\left(u_{A}\right)/\partial u dt$,

where the function *ψ_Ab_* satisfies the equation

$\psi'_{Ab}=-\partial G_{b1}\left(x_{A}\right)/\partial x$ with the boundary conditions *ψ_Ab_*(0)= *ψ_Ab_*(1).

By proceeding in a similar way one can prove that

$\frac{ds}{d\varepsilon}=\int_{0}^{1} \psi_{As}g dt$,

where the function *ψ_As_* satisfies the equation

$\psi'_{As}=-\partial G_{s}\left(x_{A}\right)/\partial x$ with the boundary conditions *ψ_As_*(0)= *ψ_As_*(1).

Similarly,

$\frac{dL}{d\varepsilon}=\int_{0}^{1} \psi_{JL}h+h \partial G_{L2}\left(u_{J}\right)/\partial u dt$,

where the function *ψ_JL_* satisfies the equation

$\psi'_{JL}=-\partial G_{L1}\left(x_{J}\right)/\partial x$ with the boundary conditions *ψ_JL_*(0)= *ψ_JL_*(1)

and

$\frac{dq}{d\varepsilon}=\int_{0}^{1} \psi_{Jq}h dt$,

where the function *ψ_Jq_* satisfies the equation

$\psi'_{Jq}=-\partial G_{q}\left(x_{J}\right)/\partial x$ with the boundary conditions *ψ_Jq_* (0)= *ψ_Jq_* (1).

After substituting the variation derivatives into *dY*/*dε* we obtain

0=*dY*/*dε*=(*α_1_+ α_12_s^*^+ α_13_p^*^+α_14_q^*^+2α_11_b_0_^*^* ) ∫_0_^1^ (*ψ_Ab_ g* +∂*G_b2_*(*u_A_*)/∂*u* *g*)*dt*+(*α_2_+ α_12_ b_0_^*^ α_23_p^*^+α_24_q^*^ +2α_22_s^*^*) ∫_0_^1^ *ψ_As_ g dt*+(*α_3_+ α_13_ b_0_^*^ +α_23_s^*^+ α_34_q^*^ +2α_33_p**) *η κ* /(*L*(*p^*^*)+*κ*)^2^ ∫_0_^1^ *ψ_JL_ h* +∂*G_L_*_2_(*u_J_*)/∂*u h dt* +(*α_4_+α_14_ b_0_^*^ +α_24_s^*^+α_34_p^*^+2α_44_q^*^*)∫_0_^1^ *ψ_Jq_ h dt.*

The functions h and g can be chosen independently, in particular, we can set each of them to zero, the above identity splits into the following two independent identities

(*α_1_+ α_12_s^*^+ α_13_p^*^+α_14_q^*^+2α_11_b_0_^*^* ) ∫_0_^1^ (*ψ_Ab_ g* +∂*G_b2_*(*u_A_*)/∂*u* *g*)*dt*+(*α_2_+ α_12_ b_0_^*^ α_23_p^*^+α_24_q^*^ +2α_22_s^*^*) ∫_0_^1^ *ψ_As_ g dt* = 0,

(*α_3_+ α_13_ b_0_^*^ +α_23_s^*^+ α_34_q^*^ +2α_33_p**) *η κ* /(*L*(*p^*^*)+*κ*)^2^ ∫_0_^1^ *ψ_JL_ h* +∂*G_L_*_2_(*u_J_*)/∂*u h dt* +(*α_4_+α_14_ b_0_^*^ +α_24_s^*^+α_34_p^*^+2α_44_q^*^*)∫_0_^1^ *ψ_Jq_ h dt=*0.

Since the functions *h* and *g* are arbitrary, we obtain

(*α_1_+α_12_s^*^+α_13_p^*^+α_14_q^*^+2α_11_b_0_^*^* ) (*ψ_Ab_* +∂*G_b2_*(*u_A_*)/∂*u*)+(*α_2_+α_12_ b_0_^*^ α_23_p^*^+α_24_q^*^+2α_22_s^*^*) *ψ_As_*=0,

(*α_3_+α_13_ b_0_^*^ +α_23_s^*^+α_34_q^*^+2α_33_p**)*η* *κ* /(*L*(*p^*^*)+*κ*)^2^ (*ψ_JL_*+∂*G_L_*_2_(*u_J_*)/∂*u)* +(*α_4_+α_14_ b_0_^*^ +α_24_s^*^+α_34_p^*^+2α_44_q^*^*)*ψ_Jq_ =*0.

We differentiate with above identities with respect to time to obtain

(*α_1_+α_12_s^*^+α_13_p^*^+α_14_q^*^+2α_11_b_0_^*^* ) (∂*G_b_*_1_(*x*_A_)/∂*x*+ d(∂*G_b_*_2_(*x'*_A_)/∂x*'*)/*dt*)+(*α_2_+α_12_ b_0_^*^ α_23_p^*^+α_24_q^*^+2α_22_s^*^*) ∂*G_s_*(*x_A_*)/∂*x* =0,

(*α_3_+α_13_ b_0_^*^ +α_23_s^*^+α_34_q^*^+2α_33_p**)*η κ* /(*L*(*p^*^*)+*κ*)^2^ (∂*G_L1_*(*x_J_*)/∂*x*+d(∂*G_L2_*(*x'_J_*)/∂*x'*)/*dt*)+(*α_4_+α_14_ b_0_^*^ +α_24_s^*^+α_34_p^*^+2α_44_q^*^*)∂*G_q_*(*x_J_*)/∂*x =*0.

This is an exact analogue of the Euler–Lagrange equations in calculus of variations providing the necessary condition of extremum.

One can see the above system of equations is strictly speaking a system of integro-differential equations since the values of *b_0_*^*^, *s*^*^, *p*^*^, *q*^*^ are given by the integration of the optimal strategies. This may seriously complicate the solution of the problem. However, one can use an iterative method of solution by fixing the values of *b_0_*^*^, *s*^*^, *p*^*^, *q*^*^ and solving the resulting system of differential equations and then plugging the obtained strategy to find *b_0_*^*^, *s*^*^, *p*^*^, *q*^*^ and then solving the system again.

Note that instead of solving the above Euler–Lagrange equations one can implement another approach (which is less general but technically easier) using some particular information about the optimisation problem. Below we show how this can be done.

Based on our numerical experiment, we firstly obtained the following approximation of the generalised fitness which corresponds to Table 1 (main text)

*Y*= 0.0278*b_0_*−0.8343*s*−1.1657*q*+33.8488*p*−0.0002*b^2^*−0.0023*bs*+ 0.0023*bq*+0.5199*bp* +0.2449*s^2^* −0.4899*sq* −3,412*sp* +0.2499*q^2^*+3.412*qp*−305.6*p^2^ .*

The Taylor expansion was obtained in the vicinity of *b_0_*=35.111, *s*=0.7316, *q*=0.3581, *p*= 0.0274.

Note that functions *x_A_*, *x_J_* are independent of each other.

Let us fix *s*, *p* and *q*. We find the derivative of the generalized fitness with respect to *b_0_*

*∂Y/∂b_0_*=0.0278−0.0004*b_0_* −0.0023*s*+0.0023*q*+0.5199*p.*

In a small vicinity of the studied point *b_0_*=35.111, *s*=0.7316, *q*=0.3581, *p*= 0.0274 we have

*∂Y/∂b_0_* ≈ 0.0278−0.0004*b_0_* – 0.0014+0.0006+0.01 ≈ 0.039 − 0.0004*b_0_*,

i.e. the derivative is positive.

Next we find the derivative of the function with respect to *p*

*∂Y*/*∂p*=33.8488+0,5199*b_0_* −3.412*s*+3.412*q*−611.2*p* ≈33.8488+18−2.1+1−611.2*p.*

One can see that in the studied vicinity the derivative is also positive.

By considering the Taylor approximation of the generalised fitness based on the quadratic form in the vicinity of the point *b_0_*=35.111, *s*=0.7316, *q*=0.3581, *p*= 0.0274, one can see that *Y* will be an increasing function of variables *b_0_* and *p*. In this case, we can fix the functional *s=C_1_* (by setting *C_1_* as a constant) and find the maximum *b_0_*^*^(*C_1_*) of the functional *b_0_*under constraint using the Lagrange multipliers method. We also fix the functional *q=C_2_*(by setting *C_2_* as a constant) and find the maximum *p^*^*(*C_2_*) of the functional *p* under constraint using again the Lagrange multipliers method. Finally, we search for the maximum of the fitness function in the form of

*Y=α_1_b_0_**(*C_1_*) *+α_2_C_1_+α_3_p**(*C_2_*)*+α_4_C_2_+α_11_b_0_*(C_1_*) *^2^+ α_12_b_0_**(*C_1_*)*C_1_ +α_13_b_0_**(*C_1_*)*p**(*C_2_*) *+α_14_b_0_**(*C_1_*)*C_2_+α_22_C_1_^2^+α_23_C_1_p**(*C_2_*)*+α_24_C_1_C_2_+α_33_p**(*C_2_*)*^2^+α_34_p**(*C_2_*)*C_2_+α_44_C_2_^2^*

by considering it as a function of two variables *C_1_* and *C_2_*.

**SM4**

Here we show an example of the construction of fitness function in model (15)-(17) in the case of a dynamic predator.

We should generally consider the space of 6 parameters characterising the prey strategy (*p*, *s*, *q*, *b_0_*, *f_A_*, *f_J_*). We will further explore the case (shown in Fig.4) where the predator only consumes juvenile prey (*f_A_* =0): note that qualitatively similar results will be obtained for *f_A_* >0 as well. The fitness function (corresponding to a single best point (strategy) in (*p*, *s*, *q*, *b_0_*, *f_J_*)) can be determined in a similar way as in (Morozov and Kuzenkov, 2016) by

$Y_{f}\left( s,p,q,b_{0},f_{A} \right)=-s-p-q-f_{A}F^{*}+\sqrt{4b_{0}p+\left( p+q+f_{A}F^{*}-s \right)^{2}}$,

where the stationary density *F^*^* of the predator can be found as the non-trivial stationary density in model (15)-(17), corresponding to the best strategy of prey (which we are intended to find using the proposed in the paper numerical algorithm).

It can be seen from the above expression that the levels curves of the fitness function *Y_f_* =const will be linear in the subspaces (*p*, *s*, *f_J_*) and (*q*, *b_0_*): these subspaces correspond to the life traits of juveniles and adults, respectively. Note that here we do not consider the situation where there are two best points (such case is impossible for the parameter domain is strictly convex, see Figs. A, B).

In the figures below (see Figs. A, B), we show the domains of feasible parameters corresponding possible combinations of *x*_A_(*t*) and *x*_B_(*t*) (technically the domains were constructed by considering various combinations of Fourier coefficients defining these functions). For further simplicity, we present the diagrams in subspaces (*p*, *s*) and (*q*, *b_0_*) since *f_J_* can be expressed as a linear function of *s*. The numerical value of *F^*^* was obtained using the numerical algorithm of the paper. Note that the best points in both figures correspond to the tangent lines of the fitness levels *Y_f_*=const to the boundary of the domain of feasible parameters of the model. The model parameters in Fig. SM is the same as in Fig.4 from the main text.


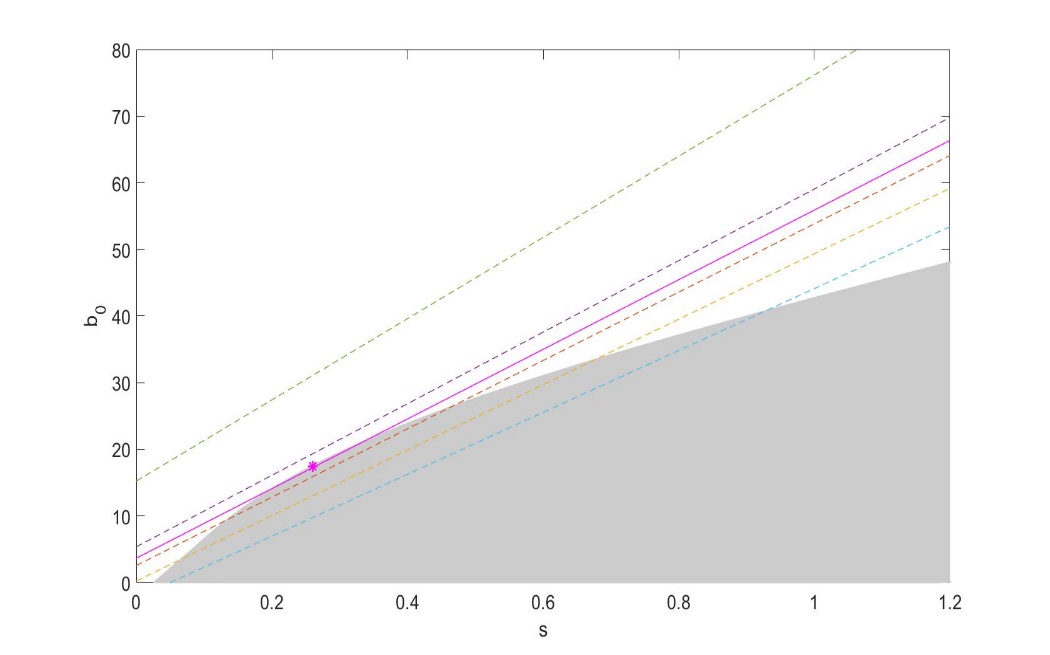


**(A)**


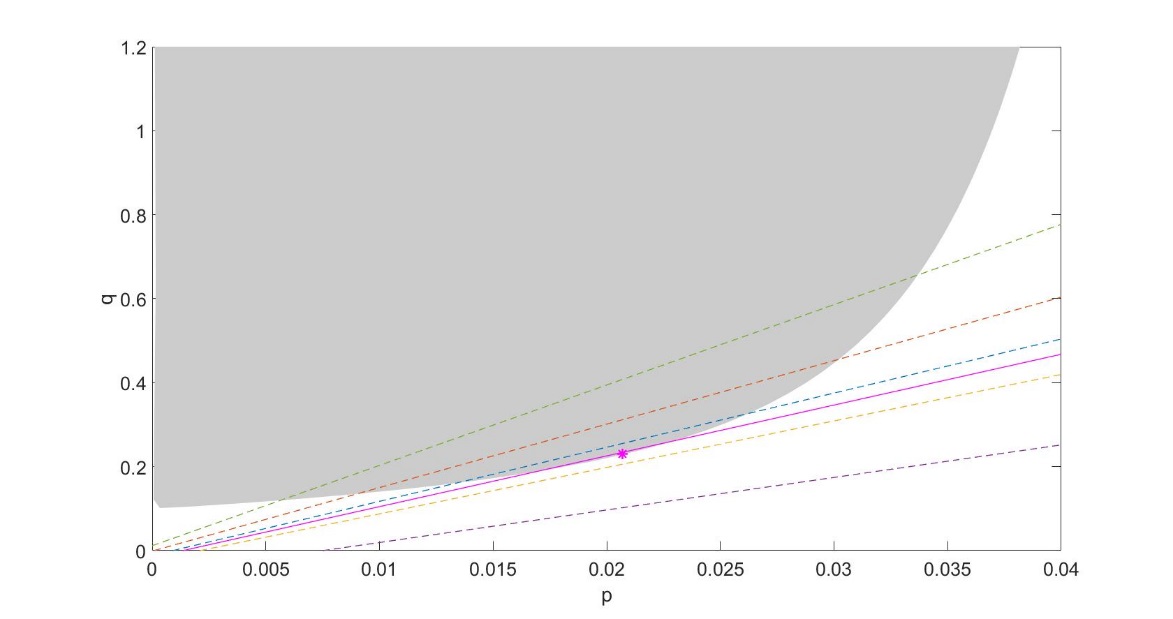


Figure SM. The levels of constant fitness *Y_f_* =const (shown by coloured lines) in the space of model parameters describing the strategies of adults (A) and juveniles (B). The domains of feasible parameters are filled in grey. The pink point in each diagram corresponds to the optimal parameters of adults and juveniles, respectively, and the pink line (tangent to the grey domain) shows the level of fitness corresponding to the best strategy. See the text for more detail.

**(B)**
